# Assessment of an *In Silico* Mechanistic Model for Proarrhythmia Risk Prediction Under the CiPA Initiative

**DOI:** 10.1002/cpt.1184

**Published:** 2018-08-27

**Authors:** Zhihua Li, Bradley J. Ridder, Xiaomei Han, Wendy W. Wu, Jiansong Sheng, Phu N. Tran, Min Wu, Aaron Randolph, Ross H. Johnstone, Gary R. Mirams, Yuri Kuryshev, James Kramer, Caiyun Wu, William J. Crumb, David G. Strauss

**Affiliations:** ^1^ Division of Applied Regulatory Science Office of Clinical Pharmacology Office of Translational Sciences Center for Drug Evaluation and Research U.S. Food and Drug Administration Silver Spring Maryland USA; ^2^ Department of Computer Science Healthcare Informatics University of Oxford Oxford UK; ^3^ Centre for Mathematical Medicine & Biology School of Mathematical Sciences University of Nottingham Nottingham UK; ^4^ Charles River Laboratories Wilmington Massachusetts USA; ^5^ CytoBioscience New Orleans Louisiana USA

## Abstract

The International Council on Harmonization (ICH) S7B and E14 regulatory guidelines are sensitive but not specific for predicting which drugs are pro‐arrhythmic. In response, the Comprehensive *In Vitro* Proarrhythmia Assay (CiPA) was proposed that integrates multi‐ion channel pharmacology data *in vitro* into a human cardiomyocyte model *in silico* for proarrhythmia risk assessment. Previously, we reported the model optimization and proarrhythmia metric selection based on CiPA training drugs. In this study, we report the application of the prespecified model and metric to independent CiPA validation drugs. Over two validation datasets, the CiPA model performance meets all pre‐specified measures for ranking and classifying validation drugs, and outperforms alternatives, despite some *in vitro* data differences between the two datasets due to different experimental conditions and quality control procedures. This suggests that the current CiPA model/metric may be fit for regulatory use, and standardization of experimental protocols and quality control criteria could increase the model prediction accuracy even further.

**Study Highlights**

**WHAT IS THE CURRENT KNOWLEDGE ON THE TOPIC?**

☑The current cardiac safety paradigm is outlined in ICH S7B and E14 guidelines, which focuses on surrogate end points (human ether‐à‐go‐go related gene (hERG) block and QT prolongation), and is highly sensitive but not very specific for predicting pro‐arrhythmic (TdP) risk.

**WHAT QUESTION DID THIS STUDY ADDRESS?**

☑Can mechanistic *in silico* models show high TdP risk prediction accuracy in a rigorous prospective study specified by the newly proposed CiPA paradigm?

**WHAT DOES THIS STUDY ADD TO OUR KNOWLEDGE?**

☑The CiPA *in silico* model and the qNet/*torsade metric score* metric demonstrate high accuracy in TdP risk prediction, which suggests it may be fit for regulatory use under the CiPA paradigm.

**HOW MIGHT THIS CHANGE CLINICAL PHARMACOLOGY OR TRANSLATIONAL SCIENCE?**

☑The established *in silico* model, together with other components of CiPA, might greatly increase the accuracy of regulatory assessment of TdP risk for clinical therapeutics.

In the 1990s to early 2000s, it was recognized that drug‐induced Torsade de Pointes (TdP), a rare but potentially fatal arrhythmia,^1^ is associated with pharmacological block of a potassium channel encoded by the human ether‐à‐go‐go related gene (hERG) and electrocardiographic QTc prolongation.^2^ This finding led to the establishment of two International Council on Harmonization (ICH) regulatory guidelines (S7B and E14) for cardiac safety assessment that focus on assessing the potential of a drug to cause hERG block and QT prolongation.^2^ Although sensitive for identifying drugs that can cause TdP, these biomarkers have low specificity.^3^ This has caused the unintended effect of deprioritizing or excluding many drugs from development that may not have actual TdP risk.^4^ In response, a new paradigm—the Comprehensive *In Vitro* Proarrhythmia Assay (CiPA)—was proposed that takes into account drug effects on multiple cardiac ion channels *in vitro* and integrates these effects into a mechanistic *in silico* cardiomyocyte model to predict TdP risk as the direct end point.^2^, ^3^, ^4^

Although numerous studies have presented *in silico* models for TdP risk prediction before,^2^, ^5^, ^6^, ^7^, ^8^, ^9^, ^10^, ^11^, ^12^, ^13^ the intended implementation of CiPA as a regulatory paradigm calls for a more stringent model qualification process. Thus, a rigorous approach was designed by the CiPA Steering Committee to strictly separate model training from validation in a stepwise manner (**Figure** 1). As a first step, 28 compounds were selected and categorized into high, intermediate, and low/no risk of TdP, and then subdivided into a training set of 12 and a validation set of 16 compounds, by a team of clinical cardiologists and electrophysiologists based on publicly available data and expert opinion.^3^ A consensus base model, the O'Hara‐Rudy dynamic (ORd) cardiac cell model,^14^ was selected by a panel of cardiac modeling experts, which then went through model optimization and metric development based on *in vitro* channel block data for the 12 training drugs.^3^ The resulting model and metric were then “frozen” and applied to the 16 validation drugs for independent validation, with the prediction outcome evaluated by predefined performance measures. This prospective design could be more stringent than the cross‐validation approach commonly used in the literature,^5^, ^6^, ^7^ and serves as a general framework for model qualification under the CiPA paradigm.

**Figure 1:**
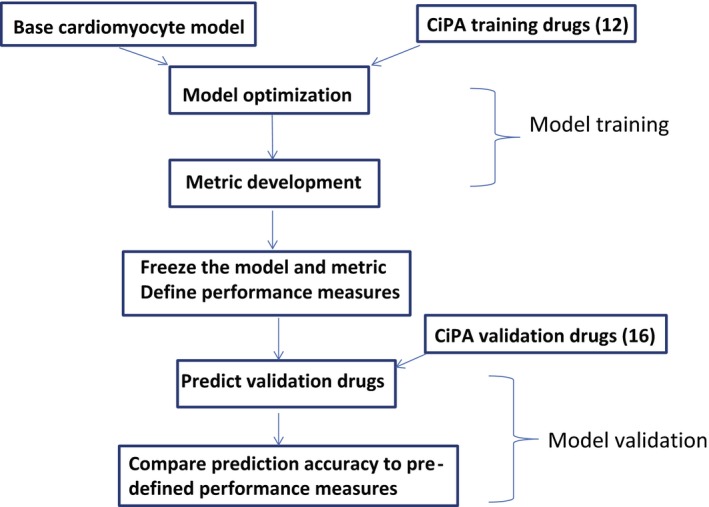
The Comprehensive *In Vitro* Proarrhythmia Assay (CiPA) *in silico* model qualification procedure. Shown is the flowchart of the CiPA *in silico* model qualification process designed by the CiPA Steering Committee. The model training process includes model optimization and metric development using published human cardiomyocyte experimental data originally used for O'Hara Rudy (ORd) model development, and newly acquired *in vitro* drug block data against various cardiac currents for the 12 training compounds. This training process was performed prior to, and strictly separated from, the model validation process—where the model and metric predefined by the training data were used to predict the TdP risk of the 16 validation drugs using their *in vitro* data. The model performance measures (**Validation Strategy/** **Supplementary Text** S1) to evaluate the prediction accuracy were also prespecified before the validation began.

Previously, we have reported the optimization of the base ORd model into CiPAORdv1.0,^15^, ^16^, ^17^, ^18^ the selection of the net charge metric “qNet” for TdP risk prediction,^15^ and the development of an uncertainty quantification method to translate experimental variability into probability distributions for the risk metric for the 12 CiPA training drugs.^17^ In this study, we report the use of this predefined modeling framework for predicting the TdP risk levels of the 16 validation drugs, to finish the model qualification process defined in **Figure** 1 and evaluate the prediction performance.

## Results

### Predefining the model, metric, and performance measures prior to validation

**Figure 2:**
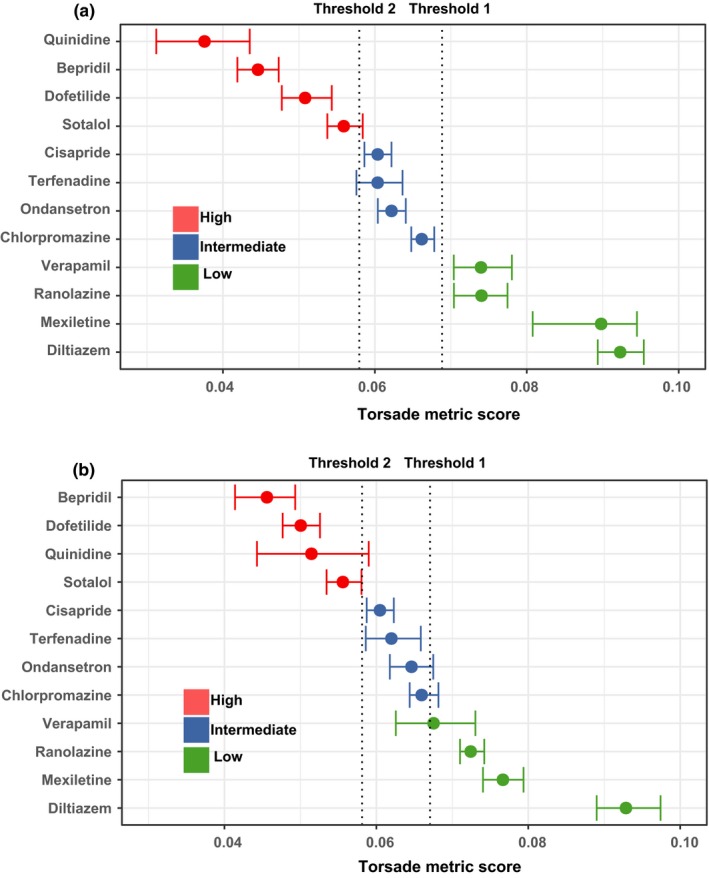
The distribution of *torsade metric scores* for the 12 Comprehensive *In Vitro* Proarrhythmia Assay training drugs. For each of the training drugs, 2,000 *torsade metric scores* were calculated using the uncertainty quantification method developed previously.^17^ The 95% confidence interval and median point of the 2,000 *torsade metric scores* for each drug are shown as horizontal error bars in this figure. Threshold 1 and threshold 2 are calculated by ordinal logistic regression (see **Supplementary Text** S2) to separate the three TdP risk categories (red: high risk; blue: intermediate risk; green: low/no risk). The values of these thresholds are given in the main text. Drugs are sorted by the median values of their *torsade metric scores* in each dataset. Results shown are for the manual (**a**) and hybrid (**b**) training datasets, respectively.

In our previous work using manual patch clamp training data, we reported that the prediction error using the pro‐arrhythmic metric qNet was lowest at 1–4 × maximum free therapeutic concentration (C_max_), where experimental data were most complete.^17^ We also concluded that three cardiac currents have the most significant impact on TdP risk prediction using qNet in CiPAORdv1.0^17^: IKr (rapidly activating delayed rectifier potassium current), INaL (late sodium current), and ICaL (L‐type calcium current). In addition, the block of INa (peak sodium current) is important for metric calculation due to its potential to cause depolarization failure.^17^ Based on these training results, we define the mean qNet value averaged across 1–4 × C_max_ as the *torsade metric score* for each drug, computed by CiPAORdv1.0 with drug effects on the four essential currents (IKr, INa, INaL, and ICaL) as model inputs. The drug effects on IKr/hERG were characterized by five dynamic parameters captured by a hERG dynamic protocol,^16^, ^19^ whereas effects on the other three currents were characterized by concentration of half inhibition (IC_50_) and Hill coefficients captured by block potency experiments.^20^ This set of model inputs^17^ was all from manual patch clamp systems at physiological temperature. The distribution of *torsade metric scores* for the 12 drugs using this manual training dataset can be found in **Figure** 2 **a**. The two thresholds that classify drugs into three TdP risk categories were also calculated and “frozen” based on the training data: Threshold 1 (separating low from intermediate/high risk) has a value of 0.0689 and threshold 2 (separating high from intermediate/low risk) has a value of 0.0579 μC/μF.

In addition, within a large‐scale multisite study that is ongoing to collect *in vitro* data for CiPA drugs using various automated high throughput patch clamp systems (HTS), one participating site (site 6) finished the block potency experiments for the 12 training drugs. As a first step to test the possibility of using automated HTS data for TdP risk assessment under the CiPA initiative, we combined site 6 HTS block potency data (IC_50_ and Hill coefficients) for INaL, INa, and ICaL with manual dynamic data for IKr/hERG to form a “hybrid” training dataset. The difference between the manual and hybrid datasets in terms of experimental conditions, and the corresponding training drug block data can be found in the validation report (**Tables 1 and 2 of** **Supplementary Text** S2). The *torsade metric score* distribution for the hybrid training dataset is shown in **Figure** 2 **b**, with the two thresholds calculated as 0.0671 and 0.0581 μC/μF, respectively.

Prior to the initiation of model validation, a comprehensive set of model performance measures (**Table 1 of Validation Strategy/** **Supplementary Text** S1) were agreed upon by the CiPA Steering Committee as well as expert electrophysiologists from the CiPA Ion Channel Working Group. The rationale of selecting these performance measures and associated acceptable levels are elaborated in the validation strategy.

### Application of the predefined modeling framework to validation drugs

Next, we applied the modeling framework defined above to the 16 validation drugs. For the IKr/hERG current, the CiPA hERG dynamic protocol^16^ was used to obtain dynamic drug‐hERG interaction parameters at physiological temperature. For non‐hERG currents (INaL, INa, and ICaL), drug block potency data were collected either through the manual patch clamp system at physiological temperature, or the site 6 automated HTS system at ambient temperature, both exactly matching the experimental conditions for the manual or hybrid training datasets, respectively. In this way, two semi‐independent validation datasets (one manual and one hybrid) were generated with shared IKr/hERG dynamic data and different non‐hERG block potency data, which were used to calculate qNet and the *torsade metric scores* for each validation drug. All the drug block parameters, as well as C_max_ used for metric calculation, can be found in the validation report (**Tables 3–5 of** **Supplementary Text** S2).

### Predefined performance measures

There are two types of performance measures for CiPA‐like risk prediction models: those evaluating the model's ability to rank order the risk levels of drugs without a specific threshold, and those using specific thresholds to classify drugs into distinct risk categories.^21^ Accordingly, the validation strategy (**Supplementary Text** S1) prespecifies two types of outcome measures for CiPA model performance evaluation: ranking performance measures and classification performance measures.

### Ranking performance measures

**Figure 3:**
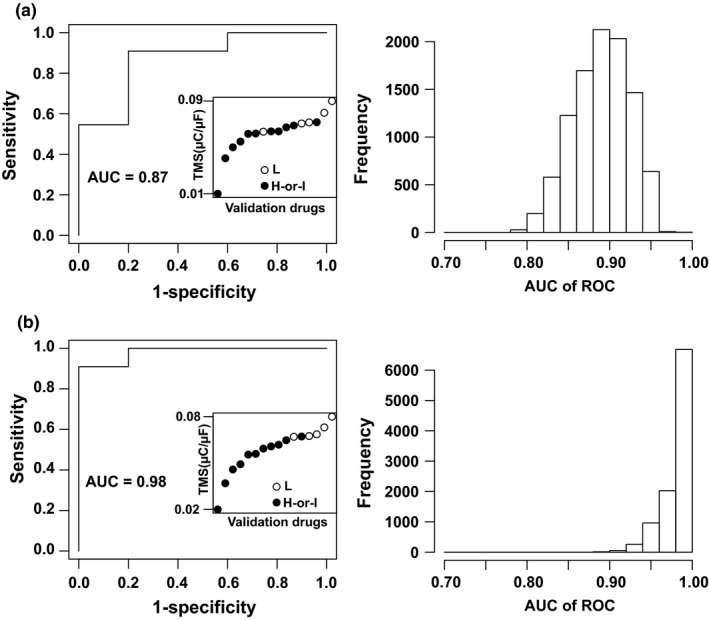
The receiver operating characteristic (ROC)1 analysis to estimate the probability of ranking high‐risk or intermediate‐risk drugs above low‐risk drugs. For ROC1 analysis, high‐risk and intermediate‐risk drugs are combined into one category (high‐or‐intermediate), and 10,000 ROC curves are constructed by sampling the *torsade metric score* distributions for the manual (**a**) and hybrid (**b**) validation datasets, respectively. Left panel of **a** or **b**: one representative example of the 10,000 ROC curves and the corresponding area under the curve (AUC). Insert: the underlying ranking of the 16 drugs (*X* axis: rank of 1–16; drug names not shown on *X* axis for figure clarity) for this particular ROC curve according to their *torsade metric score*s (*Y* axis); L: low/no risk drugs; H‐or‐I: high‐or‐intermediate risk drugs. Note that H‐or‐I drugs (black) generally have a *torsade metric score* lower than L drugs (white), indicating a higher ranking of Torsade de Pointes risk. Right panel of **a** or **b**: Distribution of the AUCs across the 10,000 ROC curves.

For the first ranking performance measure, we used all possible cutoff points along the continuous *torsade metric scores* to make binary predictions for validation drugs and assemble the results into two kinds of receiver operating characteristic (ROC) curves (high‐or‐intermediate vs. low for ROC1, and high vs. low‐or‐intermediate risk for ROC2). The area under the curve (AUC) of each ROC curve indicates the probability of correctly ranking a higher risk drug above a lower risk one.^22^ For both ROC1 and ROC2, we repeated the analysis 10,000 times by independent sampling (with replacement) from the probability distributions of *torsade metric scores* of the validation drugs. The representative ROC curves as well as distribution of AUCs across 10,000 ROC1 curves are shown for the manual (**Figure** 3 **a**) and hybrid (**Figure** 3 **b**) validation dataset, respectively. The results suggest that the probability of ranking a high‐or‐intermediate‐risk drug above (*torsade metric score* lower than) a low‐risk drug using CiPAORdv1.0 is 0.89 (95% confidence interval (CI) = 0.84–0.95) and 0.98 (0.93–1) for manual and hybrid validation datasets, respectively. Similarly, for ROC2 curves (**Figure 2 of Validation Report/** **Supplementary Text** S2), the probability of ranking a high‐risk drug above (*torsade metric score* lower than) an intermediate‐risk or low‐risk drug is 1 (0.92–1) and 0.94 (0.88–0.98) for manual and hybrid validation datasets, respectively. The median values of all these AUCs exceed or are very close to the predefined “excellent” ranking performance level (AUC > ~0.9; **Table 1 of** **Supplementary Text** S1). The second rank performance measure Pairwise Comparison, which evaluates rank performance across all three risk classes without category combining (**Validation Strategy/** **Supplementary Text** S1), also achieves predefined “excellent” performance level (>0.9) for both manual and hybrid datasets (**Figure 3 of** **Supplementary Text** S2).

### Likelihood ratio tests to evaluate classification performance

Likelihood ratio (LR) tests were prespecified to evaluate the model's ability to use predefined thresholds 1 and 2 (**Figure** 2) for TdP risk classification. For each threshold, two LRs can be calculated: LR for positive results (LR+) and LR for negative results (LR−).^23^ The resulting values (**Table 7 of Validation Report/** **Supplementary Text** S2) suggest that, using threshold 1 as a cutoff value, a high‐risk or intermediate‐risk drug is 4.5 and 8 × 10^5^ times (median of LR+) more likely to be classified into the high‐or‐intermediate category, but 8.8 and 5.5 times (median of 1/LR−) less likely to be classified into the low‐risk category, compared to a low‐risk drug, for the manual and hybrid validation datasets, respectively. Similarly, using threshold 2, a high‐risk drug is 12 and 6 times more likely to be classified into the high category, but 9 × 10^5^ and 3.7 times less likely to be classified into the low‐risk or intermediate‐risk category, compared to a low‐risk or intermediate‐risk drug, for the manual and hybrid dataset, respectively. These measures exceed or are very close to the “good” classification performance levels predefined by the Validation Strategy (LR+ and 1/LR− >~5 for good and >~10 for excellent performance).

### Mean classification error to estimate classification performance

**Figure 4:**
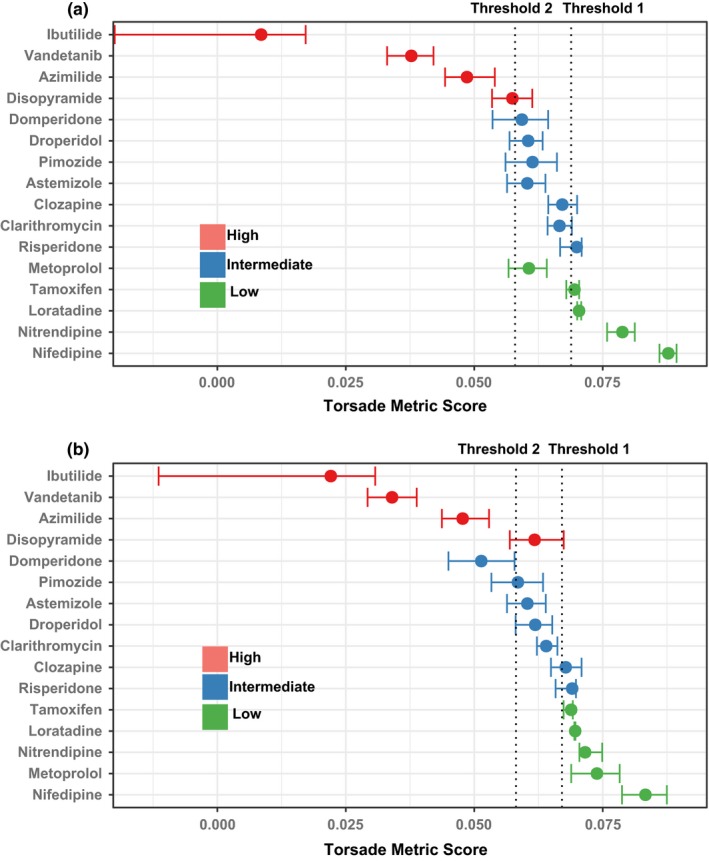
The distribution of *torsade metric score* values for the 16 Comprehensive *In Vitro* Proarrhythmia Assay (CiPA) validation drugs. For each of the validation drugs, 2,000 *torsade metric scores* are calculated using the Uncertainty Quantification (UQ) method developed earlier^17^ to describe the probability distribution of the risk metric. The 95% confidence interval and median point of the 2,000 *torsade metric scores* for each drug are shown as horizontal error bars. Drugs are sorted according to their median *torsade metric scores* within each category in each dataset. Threshold 1 and threshold 2 are predefined by training, as in **Figure** 2. (**a**) Results for the manual validation dataset. (**b**) The hybrid validation dataset.

A second classification measure (mean classification error)^7^ was prespecified to estimate the model's accuracy in classifying drugs into all three categories, which can be visualized by comparing the *torsade metric score* distributions for the 16 validation drugs to the classification thresholds (**Figure** 4). Generally, high‐risk drugs (black) have most of their distributions left of threshold 2, and low‐risk drugs (white) right of threshold 1, whereas intermediate‐risk drugs (gray) are in between.

There are some drugs that fall out of this pattern. For example, for the manual validation dataset (**Figure** 4 **a**), the intermediate‐risk drug risperidone has 75% (0.75 fraction) of its *torsade metric score* distribution classified as low risk (right of threshold 1). However, this fraction is lower than most low‐risk drugs (**Table 8 of Validation Report/** **Supplementary Text**), suggesting risperidone is still considered more dangerous than most low‐risk drugs by the model. Similarly, for the hybrid dataset (**Figure** 4 **b**), the intermediate‐risk drugs clozapine and risperidone have most of their *torsade metric score* distributions right of threshold 1. However, these distributions have the lower bounds of the 95% CI entering the intermediate‐risk zone (between threshold 1 and 2), in contrast to true low‐risk drugs, whose *torsade metric score* distributions have the entire 95% CI right of threshold 1. This suggests that these classification outliers actually arise from the use of discrete thresholds to arbitrarily cut the continuum of risk levels,^21^ which may be remedied by using ranking instead of classification for risk evaluation. Other drugs, such as metoprolol in the manual dataset, and disopyramide and domperidone in the hybrid dataset, are incorrectly predicted by both ranking and classification (outliers). Nonetheless, when summarizing all distributions of the 16 validation drugs, the mean classification error is 0.1974 (95% CI = 0.1973–0.1975) and 0.2580 (0.2579–0.2581) for the manual and hybrid datasets, respectively, both reaching the “excellent” performance level predefined by Validation Strategy (**Supplementary Text** S1).

### Some outliers may be associated with *in vitro* data discrepancy

**Figure 5:**
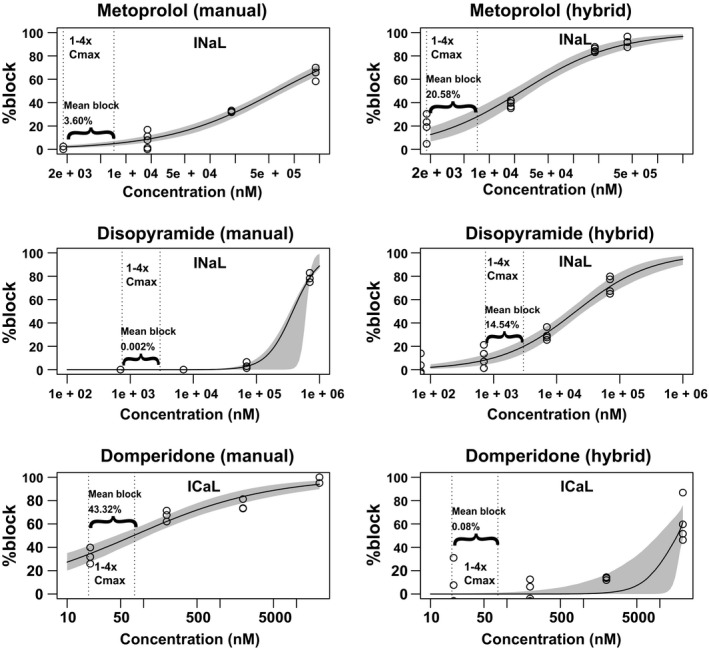
Comparison of the *in vitro* data for the outliers between the manual and hybrid datasets. The *in vitro* concentration‐dependent block data of the three outliers (incorrectly predicted by both ranking and classification) are shown for metoprolol on late sodium current (INaL) (**a**), disopyramide on INaL (**b**), and domperidone on L‐type calcium current (ICaL), and (**c**) from the manual (left) and hybrid (right) validation datasets, respectively. Circles: experimental data points for each cell. Solid line: fitting using median values of concentration of half inhibition and Hill coefficient. Gray band: 95% confidence interval of the fitting. Vertical dotted lines: the start and end of the concentration range (1–4× maximum free plasma concentration (C_max_)) used for calculating *torsade metric score*. The mean block% as estimated by the fitted Hill equation curves between 1 and 4× C_max_ is labeled. Note that for INaL block (**a** and **b**), the manual dataset shows much less potency than the hybrid dataset, whereas for ICaL (**c**), the manual dataset shows much higher potency than the hybrid dataset. This explains the emergence of outliers unique to each dataset.

The above finding that the few outliers (those incorrectly predicted by both ranking and classification) do not overlap between the two datasets raised a possibility that they might be caused by dataset‐specific experimental data bias. Because the two datasets share the same IKr/hERG data, we compared the drug block potency data on the other essential currents. Indeed, the low‐risk drug metoprolol is incorrectly classified as intermediate risk in the manual dataset but correctly predicted in the hybrid dataset (**Figure** 4), and this is associated with the fact that there seems to be an underestimation of INaL block (to offset hERG block and decrease TdP risk^24^) in the manual dataset but not the hybrid dataset (**Figure** 5 **a**). An opposite prediction pattern was seen for the high‐risk drug disopyramide (**Figure** 4), which is also associated with discrepancy in INaL block between the two datasets (**Figure** 5 **b**). Similarly, the intermediate‐risk drug domperidone is correctly predicted in the manual dataset but incorrectly predicted as a high‐risk drug in the hybrid dataset (**Figure** 4), consistent with the fact that there is a strong ICaL block to offset hERG block and suppress TdP potential^25^ in the manual dataset but not the hybrid dataset (**Figure** 5 **c**). Interestingly, these discrepancies may reflect a systematic bias between the two datasets, with most drugs' INaL IC_50s_ in the manual dataset being larger than (25 of 28 drugs) and ICaL IC_50s_ being smaller than (21 of 28 drugs) those in the hybrid dataset (**Tables 2 and 4 in** **Supplementary Text**). Preliminary results from our internal investigation suggest the different experimental conditions (e.g., use of veratridine in the manual dataset but ATX‐II in the hybrid dataset as INaL enhancers) and lack of standard quality control criteria may contribute to the *in vitro* data bias that leads to incorrect *in silico* prediction (data not shown).

### The qNet/*torsade metric score* metric outperforms alternative metrics

Next, we compared the qNet/*torsade metric score* metric to some alternative metrics reported in the literature. To examine their performance over the entire set of CiPA drugs, we combined all 28 CiPA drugs and used leave‐one‐out cross‐validation to assess the prediction accuracy. Of note, these previously suggested metrics, like APD90^7^ and APD50 plus diastolic Ca^2+^ concentration,^6^ were originally developed using different experimental protocols and simulation/statistical models, whereas here they are all calculated by CiPAORdv1.0 using either the manual or hybrid dataset. The results (**Table** 1) show that the qNet/*torsade metric score* metric generally outperforms the other ones, especially on the measures that evaluate across all three categories (pairwise comparison correct rate for ranking and mean classification error for classification). Because the drug effects on IKr/hERG were represented by binding dynamic parameters, we also evaluated two additional scenarios where simple hERG block potency was used. The first scenario uses only drug IC_50s_ in statistical equations without the need for physiological models, such as the multiple ion channel effects model^5^ and Bnet,^12^ whereas the second uses IC_50s_ and Hill coefficients for all essential currents (IKr, INaL, INa, and ICaL) in CiPAORdv1.0 to calculate metrics. Both scenarios gave worse performance than the *torsade metric score* in **Table** 1 (see **Tables 10 and 11 in Validation Report/** **Supplementary Text** S2).

**Table 1:** Prediction performance comparison using all CiPA drugs

Performance measure	Dataset	qNet/*torsade metric score*	APD90	APD50 & diastolic Ca
AUC of ROC1	Manual	0.901 (0.883–0.924)	0.842 (0.801–0.877)	0.854 (0.825–0.889)
	Hybrid	0.971 (0.936–1)	0.848 (0.807–0.889)	0.854 (0.807–0.906)
AUC of ROC2	Manual	0.988 (0.95–1)	0.975 (0.962–0.988)	0.988 (0.944–1)
	Hybrid	0.919 (0.869–0.962)	0.975 (0.956–0.981)	0.969 (0.925–0.981)
Pairwise comparison correct rate	Manual	0.929 (0.905–0.943)	0.886 (0.858–0.91)	0.891 (0.829–0.924)
	Hybrid	0.943 (0.905–0.976)	0.891 (0.863–0.919)	0.896 (0.858–0.929)
LR+ of threshold 1	Manual	8.05 (4.03–9)	2.53 (1.89–2.84)	4.03 (2.68–4.26)
	Hybrid	8.05 (4.03–9.47e+05)	2.68 (2.01–4.03)	3.55 (2.37–4.26)
LR− of threshold 1	Manual	0.0677 (1.13e−06–0.178)	0.189 (0.0789–0.316)	0.135 (0.0677–0.203)
	Hybrid	0.0677 (1.12e−06–0.158)	0.158 (0.0789–0.284)	0.203 (0.0789–0.316)
LR+ of threshold 2	Manual	7.5e+05 (8.75–1e+06)	15 (12.5–17.5)	17.5 (15–8.75e+05)
	Hybrid	15 (6.25–17.5)	15 (12.5–17.5)	15 (7.5–17.5)
LR− of threshold 2	Manual	0.25 (1e−06–0.263)	0.263 (0.132–0.395)	0.25 (1.05e−06–0.263)
	Hybrid	0.263 (0.132–0.395)	0.263 (0.132–0.395)	0.263 (0.132–0.395)
Mean classification error	Manual	0.158 (0.155–0.161)	0.305 (0.301–0.309)	0.224 (0.221–0.228)
	Hybrid	0.203 (0.201–0.208)	0.285 (0.281–0.289)	0.291 (0.287–0.295)

APD, Action Potential Duration; AUC, area under the curve; Ca, Calcium; CiPA, Comprehensive *In Vitro* Proarrhythmia Assay; LR, likelihood ratio; ROC, receiver under the curve. For each performance measure (row), the values for three metrics (qNet/*torsade metric score*, APD90, APD50 and diastolic Ca^2+^) using the two datasets (manual and hybrid) are shown. All 28 CiPA drugs are used, with leave‐one‐out cross‐validation to calculate the performance measure. The median as well as 95% confidence interval values for each performance measure are listed.

## Discussion

In this report, we documented the prediction performance of the CiPAORdv1.0 model on the 16 CiPA validation drugs, using model, metric, and classification thresholds predefined by the 12 CiPA training drugs, and performance measures specified prior to the validation. Adopting a principle similar to a clinical trial design,^26^ this CiPA model qualification strategy is the most stringent approach so far to evaluate a TdP risk predictor. Even though a particular model (CiPAORdv1.0) was used in this study, this qualification process could be generally applied to any model to evaluate its fitness for regulatory use under the new CiPA paradigm.

The prespecified performance measures evaluate the accuracy of both ranking and classification. It has been suggested that, for biomarker‐based risk prediction on individual patients, ranking along the continuous metric score may be a more realistic representation of the risk than specific threshold‐based classification.^21^ Even though the end point of the CiPA model is a drug's overall TdP risk in the general population rather than on a specific patient, the same rationale may apply. Besides, although ranking is solely based on a physiological metric (qNet) that is inherent to each drug's electrophysiological properties, classification thresholds are established by statistical models and, thus, dependent on other drugs in the training set. With the relatively small training set,^12^ we expected a better performance using the ranking measures than classification. The results are consistent with this hypothesis, and some drugs (e.g., risperidone) are indeed incorrectly predicted by classification but not ranking. Nevertheless, across two validation datasets, the ranking performance measures (five excellent and one good) and classification performance measures (five excellent, three good, and two minimally acceptable) are all above the prespecified acceptable performance levels. Because these predefined performance levels are arbitrary, we also compared qNet/*torsade metric score* to alternative metrics, and found a general superior performance (**Table** 1 and Validation Report). Due to the difference in drug block data used and the underlying mathematical models, this is a performance comparison within the CiPA framework, not a direct examination of the accuracy of the original predictors. This partly explains why the measures from some alternative metrics in **Table** 1 are below those originally reported, although the omission of INaL block data when developing these original metrics^5^, ^6^, ^7^ may also contribute to their suboptimal performance with CiPA validation drugs, given that a drug's significant block on INaL may balance off its hERG block effect and reduce its torsadogenic potential.^24^

Some drugs are incorrectly predicted in at least one of the validation datasets. One possible reason is these drugs' complex pharmacokinetic or pharmacodynamic effects are not adequately captured by the model. For example drug‐drug interaction is suggested to play a role in torsadogenic potential of antipsychotics, such as clozapine and risperidone,^27^ and antiemetics like domperidone.^28^ In addition, it has been suggested that risperidone's active metabolite paliperidone plays a more clinically important role in disturbing repolarization than the parent drug.^29^ The actual clinical free plasma concentration of disopyamide is subject to not only drug‐drug interaction^30^ but also concentration‐dependent protein binding.^31^ In addition, some beta‐blockers, like metoprolol, may have reduced torsadogenic liability through counteracting adrenergic‐mediated TdP effect^32^ and protecting against dispersion of repolarization.^33^ Because the model produced mixed results for most of these drugs (some correctly predicted by ranking but not classification; others correctly predicted in one dataset but not the other), it is difficult to estimate to what degree the omission of these pharmacological effects in the model negatively impacted the prediction. Further studies may be needed to elucidate this.

Another possible reason for incorrect prediction is dataset‐specific mischaracterization of *in vitro* data, as suggested by the association between the emergence of dataset‐specific outliers and the systematic *in vitro* data discrepancy between the two datasets (**Figure** 5). Indeed, the two datasets used quite different experimental conditions on many of the currents (**Table 1 of** **Supplementary Text** S1), and our own investigation suggests suboptimal quality control procedures (i.e., lack of control for seal resistance, baseline stability, etc.) could have affected some specific IC_50_ data from both the manual and hybrid datasets, which led to the systematic data discrepancy between them. A standard operating procedure and unified quality control criteria are being established under the CiPA initiative, which could increase the model prediction accuracy even further.

Although the current model and metric were validated by two semi‐independent *in vitro* datasets sharing the same IKr/hERG data, additional truly independent data from different laboratories will further test the reproducibility of the *in vitro* data. This may enable the development of *in silico* methods to adjust laboratory‐to‐laboratory variabilities so that all new drugs can be compared to the same set of reference drugs and thresholds. In addition, the adoption of CiPA in the early discovery stage would be greatly facilitated by adjusting the model and/or experimental protocols so that the hERG data can be collected by automated high throughput patch clamp systems at ambient temperatures. Nevertheless, the TdP assessment framework described in this report has high accuracy for proarrhythmia risk assessment, suggesting it may be fit for CiPA's intended purpose of supporting regulatory decision making.

## Methods

Detailed methods are published previously and/or detailed in **Supplementary Text** S2, with only a few critical aspects of the procedures highlighted here.

### Experimental procedure

The dynamic hERG protocol^16^ and manual non‐hERG protocols^20^ were published previously. For the hybrid dataset, the block on non‐hERG currents were measured using CiPA ion channel working group protocols, detailed in **Supplementary Text** S2.

### Simulation procedure

The CiPAORdv1.0 model,^17^ including the dynamic hERG submodel,^16^ as well as the qNet metric,^15^ and the uncertainty quantification method to estimate probability distribution and define classification thresholds through ordinal logistic regression,^17^ were all previously published. All the software is available at https://github.com/FDA/CiPA.

### Performance measures

All performance measures were predefined in the Validation Strategy (**Supplementary Text** S1). The probability distribution of *torsade metric scores* for each drug was randomly sampled with replacement 10,000 times to estimate the median and 95% CI values of performance measures.

## Funding

This work was supported by the Wellcome Trust (grant number 101222/Z/13/Z) and by the Engineering and Physical Sciences Research Council (grant number EP/G037280/1). The Wellcome Trust grant jointly with The Royal Society through a Sir Henry Dale Fellowship awarded to G.R.M. R.H.J. was supported by a Systems Approaches to Biomedical Science Industrial Doctorate Centre studentship by the UK EPSRC and F. Hoffmann‐La Roche AG. This project was supported by the Research Participation Program at CDER, administered by the Oak Ridge Institute for Science and Education (ORISE) through an interagency agreement between the US Department of Energy and the US Food and Drug Administration.

## Conflict of Interest

Gary Mirams has received research support from consultancy to Oxford University Innovation on projects with Hoffman‐La Roche and GlaxoSmithKline. This report is not an official US Food and Drug Administration guidance or policy statement. No official support or endorsement by the US Food and Drug Administration is intended or should be inferred.

## Author Contributions

Z.L. wrote the manuscript. Z.L., D.G.S., and G.R.M. designed the research. Z.L., G.R.M., R.H.J., X.H., M.W., J.S., P.T., A.R., W.W., Y.K., J.K., C.W., W.C., and B.J.R. performed the research.

## Supporting information


**Supplementary Text S1**. CiPA *in silico* model validation strategy.

**Supplementary Text S2**. CiPA *in silico* model validation report.
