# Supplementary material for: Assessment of an In Silico Mechanistic Model for Proarrhythmia Risk Prediction Under the CiPA Initiative
Source: Clin Pharmacol Ther. 2018 Aug 27;105(2):466–75. doi: 10.1002/cpt.1184 (PMC6492074; doi:10.1002/cpt.1184)
Supplement: Supplementary file 1 — Supplementary Text S1. CiPA in silico model validation strategy. [file CPT-105-466-s001.pdf]

CiPA *In Silico* Model Validation Strategy

CiPA *In Silico* Working Group

11/27/2017

1. Executive Summary

Within the Comprehensive In Vitro Proarrhythmia Assay (CiPA), a candidate computational cardiomyocyte model (CiPAORdv1.0) and associated mechanistic metric (qNet) have been developed to define a Torsades Metric Score (TMS) based on CiPA training dataset describing the effects of 12 drugs on multiple cardiac currents in heterologous expression systems. We propose to validate the model and metric with 16 additional drugs using two validation datasets (VDs), namely 1) VDMANUAL, consisting of hERG dynamic data and non-hERG block potency data from manual patch clamp systems, and 2) VDHYBRID, consisting of hERG dynamic data generated from manual patch clamp systems as well as non-hERG block potency data obtained from automated High Throughput Systems (HTS). Both validation datasets will be used to evaluate the performance of TMS based on its performance on two separate tasks: (a) the ability to rank order the validation drugs according to their relative TdP risk, and (b) the ability to classify each validation drug into one of the three TdP risk categories (High, Intermediate, and Low/No) using classification thresholds derived from the training data set. These two validation endpoints correspond to distinct scenarios for applying TMS to regulatory and/or drug development decision making and will define the role of *in silico* modeling as a regulatory paradigm in CiPA. Model performance assessment measures

and acceptable performance levels are pre-defined to ensure an objective and comprehensive evaluation of the predictive power of the CiPA *in silico* model.

2. Summary of the Training Phase

The CiPA *In Silico* Working Group (ISWG) completed the development of a computational cardiomyocyte model and associated mechanistic metric for assessing a drugs' TdP risk level based on 12 CiPA training compounds. The most recently published Optimized IKr-dyn ORd model [1] has been "frozen" as CiPAORdv1.0. This model and the candidate metric (qNet) were developed and calibrated using manual patch clamp data for the 12 CiPA training compounds [1]. Specifically, dynamic drug-IKr (rapidly activating delayed rectifier potassium current) interaction was characterized with the CiPA dynamic hERG protocol (modified from Milnes protocol) at physiological temperature by Dr. Wendy Wu's lab at FDA [2], and drug block potency data for the other cardiac currents were collected using Dr. William Crumb's protocols at physiological (L-type Ca^{2+}/ICaL, late sodium/INaL, fast sodium/INa, slowly activating delayed rectifier potassium current/IKs, and inward rectifier potassium current/IK1) and room (transient outward potassium current/Ito) temperature, respectively [3]. All these data were integrated into CiPAORdv1.0, and the qNet value was computed as the net charge carried by six of the major cardiac ionic currents (IKr, IKs, Ito, IK1, ICaL, and INaL). A method was also developed to incorporate experimental data uncertainty into the model simulation and risk evaluation process [4]. The resulting model and metric can rank the training compounds into a correct order (qNet values of Low Risk drugs > those of Intermediate > those of High Risk drugs) from 1 to 25x maximum free therapeutic plasma concentrations (Cmax) at fast (120 beats per minute), normal (60 beats per minute), and slow (30 beats per minute) [1] heart rates. There is a pattern for the risk

category separation to be better at higher drug concentrations and slower heart rates (see Fig 4 and Fig 5 in [1]). However, uncertainty quantification analysis indicates that at too high a concentration (>= 5x Cmax) the high experimental data uncertainty would translate into a high uncertainty in the qNet value and risk level prediction [4]. To keep a balance between optimal risk stratification and reliable metric calculation, a decision was made to use the mean qNet values averaged across clinically relevant concentrations (1x, 2x, 3x, and 4x Cmax) at a cycle length of 2000 ms (heart rate 30 beats per minute to mimic bradycardia) as each drug's characteristic TdP risk metric value, termed Torsades Metric Score (TMS). A rank order of the TMS for all 12 CiPA training drugs after considering experimental uncertainty is illustrated in **Fig 1A**.

In addition to manual data, automated patch clamp data using CiPA step protocols from automated High Throughput Systems (HTSs) were also collected and tested in the model. It was found that the HTS platforms did not produce dynamic hERG-drug interaction data of sufficient quality in the HESI-coordinated study to be used in the model. So at this stage a combination of manual hERG dynamic data (Dr. Wendy Wu's lab at FDA) and automated HTS drug block potency data (IC50s) for non-hERG currents were used. Among the various HTS sites participating in the HESI study, only one site (site 6) submitted complete data for all 12 training drugs. Subsequent modeling shows that it is possible to use manual hERG dynamic data (produced at physiological temperature) and site 6's non-hERG HTS data (produced at room temperature) to rank order the 12 CiPA training compounds (**Fig 1B**).

Fig 1. Torsades Metric Score (TMS) distribution of training data.

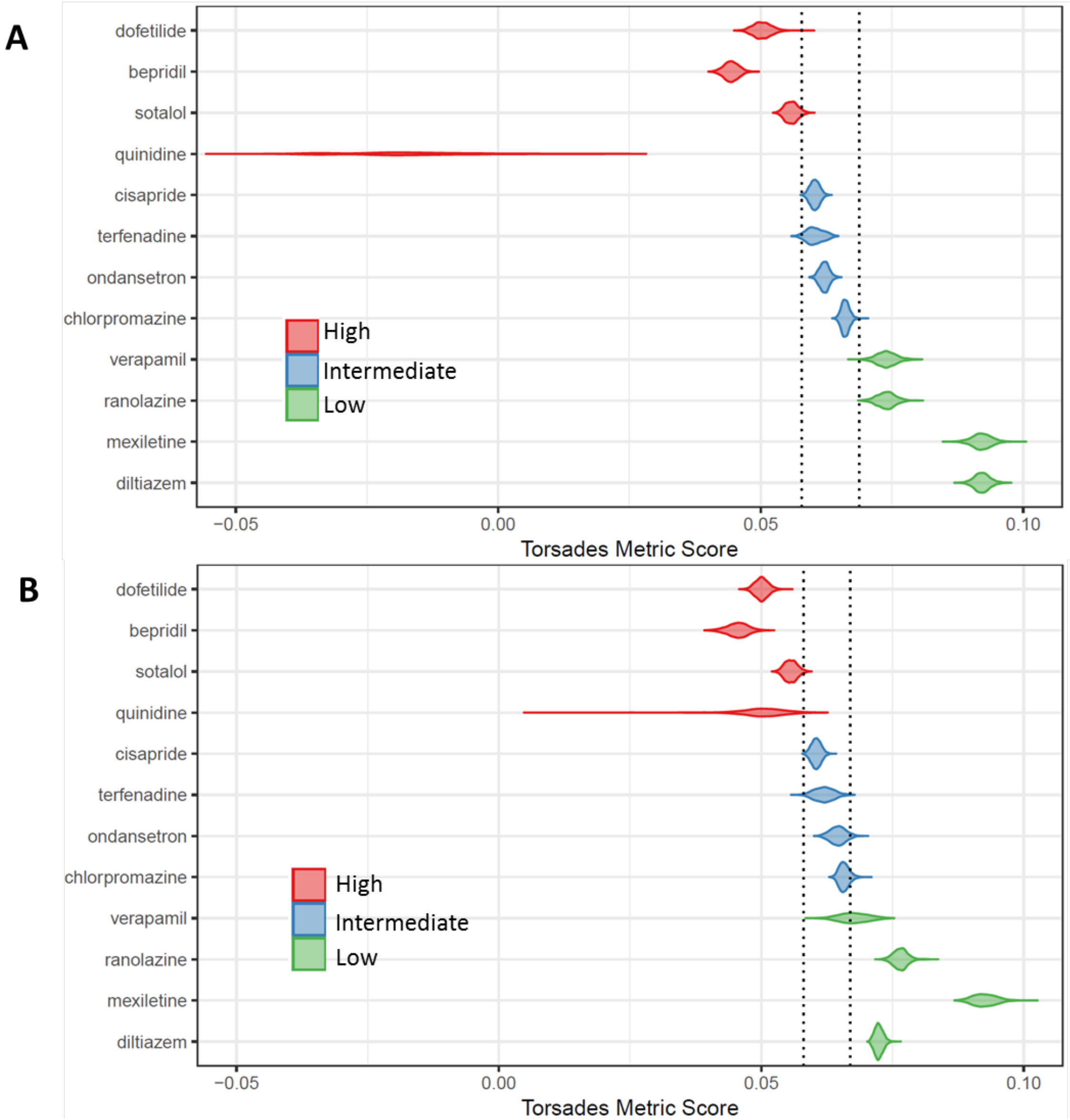

A. Distribution of TMSs (qNet averaged from 1 to 4x Cmax) for the 12 training drugs using manual patch clamp derived data (CiPA manual dynamic protocol for hERG and Dr. William Crumb's manual patch clamp protocols for non-hERG channels). For each drug, the distribution of the 2000 TMS values is shown as a density plot (cloud), with the thickness of the cloud proportional to the relative probability for that drug to have a specific TMS value as on the X axis. The two dotted lines are the two thresholds separating High, Intermediate, and Low risk

categories, established by ordinal logistic regression (see text for details). **B**. The same plot for the 12 training drugs using hybrid data (manual CiPA dynamic protocol for hERG and site 6's HTS automated data using CiPA protocols for non-hERG channels).

3. Validation Strategy

3.1 Model and Metric

The recently published model (CiPAORdv1.0) and the metric (qNet) will be used. For each of the 16 validation drugs, the mean qNet value averaged across 4 concentrations (1x, 2x, 3x, and 4x Cmax) at a cycle length of 2000 ms will be used to characterize the TMS. Using the established uncertainty quantification method [4], a probability distribution of the TdP risk metric characterized by 2000 TMS values will be generated for each drug. Note that here we use 2000 TMS values per drug to keep the validation consistent with the training stage. For reduced computational complexity and ease of implementation we may try to find a minimally acceptable number per drug that can give a reasonable approximation of the TMS probability distribution after the validation stage.

3.2 Dataset

As with the training data, two types of datasets will be collected for the 16 validation compounds. One type is a pure manual dataset (termed VDMANUAL), with the hERG dynamic data collected by CiPA dynamic protocol in Dr. Wendy Wu's lab at FDA and non-hERG drug potency data collected by Dr. Willam Crumb's protocols at his laboratory. In this manual dataset drug effects on only 4 (IKr, INaL, INa, and ICaL) out of 7 cardiac currents will be collected, because uncertainty modeling suggests that all other currents have minimal impact on TdP risk assessment in CiPAORdv1.0 [4]. For VDMANUAL all data are to be generated at physiological temperature. The other type is a hybrid dataset (termed VDHYBRID), with the hERG dynamic data from Dr. Wendy Wu's lab as above and non-hERG potency data using standard CiPA protocols from automated HTS sites participating in the study. In this hybrid dataset drug effects on all 7 cardiac currents will be collected, but initial validation will be performed using only 4 currents' data to keep it consistent with VDMANUAL. For VDHYBRID the hERG dynamic data are generated at physiological temperature, while non-hERG block potency data will be obtained at either physiological or room temperature, depending on the participating site's configuration. In addition to these two main datasets, a pure HTS dataset that contains both hERG and non-hERG drug block potency (IC50s) data from HTS platforms can be put through the validation pipeline for comparison purpose. This dataset is not the main validation data because our previous studies [1, 2] have shown that ignoring drug-hERG dynamic interactions will negatively impact the *in silico* TdP risk prediction precision. However, including this dataset can test the boundary conditions of the CiPA *in silico* model in terms of input data quality and accuracy and help to establish the *in vitro* data standards for CiPA implementation.

3.3 Validation strategy

Among the many possible measures to evaluate the performance of a predictive model [5], the most commonly used ones belong to two types: those evaluating the model's ability to rank order the validation cases (drugs) without a specific threshold (cutoff of the continuous metric), and those using specific thresholds to classify the validation cases into distinct groups (i.e. TdP risk levels) [5]. Accordingly, the TMS will be evaluated by these widely used measures in relation to the predictive performance of two tasks: rank order the validation drugs, and classify the validation drugs.

3.3.1 Evaluation of the ranking order capability

For a predictive model outputting a continuous metric score like the CiPA model and qNet metric, instead of establishing specific classification thresholds based on the limited training data, one could try all possible thresholds and apply each of them to the validation data to calculate a series of sensitivity and specificity values. A plot of sensitivity vs 1-specificity defines Receiver Operating Characteristic (ROC) curve, which summarizes the overall performance of the model/metric, with the Area Under the Curve (AUC) of ROC, or c (concordance) statistic, indicating the model's rank order capability [6]. One of the greatest strengths of such a measure is its interpretability: the AUC of ROC is the probability for the model to correctly rank order a pair of cases (drugs), each from a distinct group (risk category). Based on non-parametric Mann-Whitney U statistics [7], this rank order statistic gives a meaningful and intuitive interpretation of the predictive power of the model. In fact it has been argued that such a rank order-based statistic is preferred over threshold-based classification in evaluating a predictive model with continuous output metric [5].

Traditionally ROC analysis is applied to dichotomous prediction, i.e. the predicted outcome is either high or low risk. This is because the concepts of sensitivity (e.g. proportion of true high risk drugs predicted to be high risk) and specificity (e.g. proportion of true low risk drugs predicted to be low risk) were developed for binary predictions. There are theoretical frameworks to extend ROC to trichotomous predictions [8], when the outcome could be high, intermediate, or low risk as in CiPA. However, because this extension is not widely used, we decided to use the traditional two-way ROC, and convert the three-way CiPA categorization into two-way by combining two adjacent categories into one. Thus two separate rounds of ROC analysis will be performed: the first round combining intermediate with high risk while the second round combining the intermediate with low risk. The AUCs of ROC for these two rounds of analysis have easily interpretable meaning: the first AUC is the probability for this model to rank a low risk drug's qNet value above a high-or-intermediate drug (the higher qNet value the safer), while the second is the probability to rank a high risk drug's qNet value below an intermediate-or-low risk drug. From a regulatory perspective the ability to distinguish between low vs intermediate-or-high risk drugs is more important, and hence the first AUC should get more weight in evaluating the overall performance of the model/metric.

There are no absolute rules about how large an AUC is needed for the predictive model to be useful. Generally, because a random guess would have a ranking probability (AUC of ROC) of 0.5, models with AUCs below 0.6 are not considered useful [5]. For CiPAORdv1.0 to be considered usable, we propose three levels of acceptable performance based on AUC values: a minimally acceptable performance of >~0.7, a good performance of >~0.8, and an excellent performance of >~0.9 (Table 1). These three levels of AUCs are generally considered to be linked with

good, very good, and excellent diagnostic accuracy, respectively [9]. It is worthwhile to point out that some studies reported very high AUCs (>0.93) in their model's ability to separate high TdP risk drugs from low risk ones [10, 11]. However, due to the difference in drug selection (many studies lack balanced hERG/INaL blockers like ranolazine), in TdP risk categorization systems (3-way categories vs 2-way), in the nature of the metric (single mechanistic metric vs using a statistical model to combine multiple metrics), and in the validation stringency (dedicated validation set vs leave-one-out cross-validation), the AUCs for different models from other studies should not be compared directly to those for CiPAORdv1.0, and the proposed acceptable performance levels should be considered specific for CiPA validation study.

Because the ROC analysis proposed above evaluates only the ranking performance within the validation dataset and involves reducing the three CiPA categories into two, we will also perform two more tests to measure the model/metric's ability to rank a drug relative to all 28 CiPA drugs across all 3 categories. In the first test, each of the 16 validation drugs will be ranked against the whole CiPA drug list (the lower the qNet value, the higher the risk rank). The 2000 TMS values for each drug will give rise to 2000 rankings, the distribution of which reflects the stability of that validation drug's relative predicted TdP risk. In the second test, the three categories of all 28 CiPA drugs will be decomposed to a series of pairwise comparisons/rankings. Out of the 378 (28*27/2) theoretical pairwise combinations, drug pairs where both compounds are from the same category (so the exact ranking is unclear), or both compounds are from the training set (so the information has already been used for model building), will be removed, giving rise to 211 validation pairs. The percentage of correctly predicted pairwise rankings among the 211 pairs gives an estimation of

the model/metric's overall ranking performance across the full set of CiPA reference drugs. Since there are no established criteria with regard to the relationship between pairwise ranking performance and diagnostic accuracy, we propose to use the same three levels of acceptable pairwise ranking performance as AUC, namely a minimally acceptable performance of >~0.7, a good performance of >~0.8, and an excellent performance of >~0.9 (Table 1). Importantly, even though the full list of 28 CiPA drugs will be used, both the two tests measure the model's ability to rank a new compound (validation drug) not used in training, thus should still be considered independent validation.

3.3.2 Evaluation of the classification capability

The second measure evaluates the CiPA model's ability to classify the validation compounds into one of the three TdP risk categories (high, intermediate, low) by establishing thresholds (cut-points) of the continuous qNet value based on the training data. The two thresholds, one (Threshold 1) separating low from intermediate/high risk and the other (Threshold 2) separating low/intermediate from high risk, will be established by applying ordinal logistic regression to the training data (**Fig 1**), similar to the cross-validation study in the uncertainty quantification analysis [4]. Statistically, Threshold 1 is the cut-off point of qNet values where a drug has 50% probability of being a low risk drug and 50% probability intermediate-or-high risk drug, while Threshold 2 is the cut-point having 50% probability of being low-or-intermediate and 50% probability of being high risk. As a technical note, ordinal logistic regression usually assumes the input data are independent from each other, while with CiPA's uncertainty dataset each drug's 2000 TMS values are clearly correlated. One widely used method to handle correlated data for ordinal logistic regression is Generalized Estimating Equations (GEE) [12-14], however the

enormous amount of data (2000 per drug) are beyond the processing capacity of any existing implementation of this method (Dr. Touloumis and Dr. Parsons, personal communications). Using small samples of the uncertainty data we found that modeling the internal correlation through GEE had minimal effect on the calculated thresholds, so for the full analysis we treat each data point as independent and apply ordinal logistic regression to the entire dataset. It is worth reiterating that the whole classifier (the ordinal logistic regression equation, Threshold 1, and Threshold 2) are established solely based on the 12 training drugs, after which they will be “frozen” and then used to predict the risk categories of 16 validation drugs in an independent manner.

For classification of each of the validation compounds, the ordinal logistic regression equation established from training data will be applied to each of the 2000 TMS values and predict the probability of this TMS value belonging to each one of the three risk categories. This TMS value is then assigned to the risk category with the highest probability. Statistically this is equivalent to comparing each of the TMS values to the two thresholds (Threshold 1 and 2) described above. A TMS value above Threshold 1 will be assigned to Low Risk, while a TMS value between the two thresholds will be assigned to Intermediate Risk, and finally a value below threshold 1 to High Risk.

To evaluate the classification performance, we will adopt the widely used diagnostic Likelihood Ratio (LR) as a measurement [15]. Similar to the ROC analysis used for ranking performance evaluation, we will conduct two rounds of LR analysis, first evaluating the model’s ability to use Threshold 1 to classify drugs as Low vs Intermediate-or-High risk, and then use Threshold 2 to classify drugs as Low-or-

Intermediate vs High risk. For each round of LR analysis, two LR values will be calculated: LR+ ($sensitivity/(1-specificity)$) for positive prediction and LR- ($(1-sensitivity)/specificity$)) for negative prediction. This will give rise to a total of 4 LR calculations that directly assesses the prediction accuracy. LR+ for first analysis indicates how much more likely an Intermediate-or-High risk drug would have TMS below Threshold 1 (thus classified correctly) compared to a Low risk drug. LR- for first analysis indicates how much less likely an Intermediate-or-High risk would have TMS above Threshold 1 (thus classified incorrectly) compared to a Low risk drug. Similarly, LR+ for second analysis indicates how much more likely a High risk drug would have TMS below Threshold 2 (thus classified correctly) compared to an Intermediate-or-Low risk drug, while LR- for second analysis indicates how much less likely a High risk drug would have TMS above Threshold 2 (thus classified incorrectly) compared to an Intermediate-or-Low risk drug. Because specific pre-defined thresholds are used for classification, this type of analysis evaluates the performance of classification rather than ranking.

Generally, a diagnostic model or test with LR+ above 2, 5, and 10 (correspondingly LR- below 0.5, 0.2, and 0.1) is considered to have small (but sometimes important), moderate, and large predictive power, respectively [15]. Accordingly we propose to use these 3 levels as the benchmark for minimally acceptable, good, and excellent performance of our model in the validation study (Table 1). While other studies have developed TdP risk prediction models and reported LRs [10], the different ways of selecting drugs, defining risk levels, and performing validation dictate that the acceptable LR performance defined here is specific for the CiPA validation study and the LRs generated by CiPAORdv1.0 are not directly comparable to other study's [9]. For the first round of LR analysis (classifying Low vs Intermediate-or-High), the

performance of LR+ may carry more weight since the primary goal of CiPA is to increase the specificity of positive test (predicting a drug has a risk above the Low category) [16]. For the second round (classifying Low-or-Intermediate vs High), LR- may be favored because true High risk drugs are relatively rare and may only be readily detectable by a model with high sensitivity.

The LR analysis proposed above is widely used to evaluate the diagnostic performance of a model/test when the outcome is binary, but could not assess the model performance using two thresholds (Threshold 1 and Threshold 2) for 3-way classification simultaneously. To summarize the overall classification performance, we propose to adopt an error scoring system as in Mirams et al. [17]. Briefly, denoting Low Risk as category 0, Intermediate Risk as category 1, and High Risk as category 2, for each TMS value the classification error = | predicted category – true category |. The mean error across the 2000 TMS values represents that drug's classification error after considering experimental uncertainty. And the mean of the classification errors across 16 validation drugs indicates on average how far the prediction deviates from the true category.

There is no standard as to how low the error needs to be for a classifier to be useful. For reference, in Mirams et al. the best metric APD90 had an average error of 0.323, while two other metrics hERG IC50 and safety margin (hERG IC50/free plasma concentration) had errors of 1.129 and 0.968, respectively. Since the CiPA *In Silico* validation strategy of using a dedicated validation dataset is more stringent than the leave-one-out cross validation strategy used by Mirams et al. we propose setting a minimally acceptable performance level of error < ~1 (to match the second best classifier in Mirams study), and an excellent performance level of error < ~0.3 (to

match the best classifier in Mirams study), with a good performance level set at an arbitrary value 0.5 in between (Table 1). On the other hand, given that virtually all *in silico* TdP risk assessment studies used leave-one-out cross validation due to the small number of drugs [10, 11, 17], we will also perform a leave-one-out cross validation using all 28 CiPA drugs and compare the prediction error to that of the dedicated 16 validation drugs, to evaluate how much of the error is due to the random separation between the training (12) and validation (16) compounds.

3.4 In Relation to Implementation

The two validation datasets (VDMANUAL and VDHYBRID) and two performance measures (ranking capability and classification capability) give rise to four validation endpoints (**Fig 2**). Each of the four endpoints corresponds to a unique scenario of using the model for regulatory and/or drug development decision making. A good ranking capability or a good classification capability of the TMS using VDMANUAL suggests they could be used to rank order a new compound's TdP risk relative to reference CiPA compounds or predict a new compound's TdP risk category respectively, if the new compound's *in vitro* ion channel data were collected in a way consistent with VDMANUAL (CiPA dynamic protocol for hERG and Dr. William Crumb's protocols for non-hERG channels at physiological temperatures). Similarly a satisfying ranking capability or classification performance of TMS using VDHYBRID supports the use of them to rank a new compound or predict the TdP risk category of a new compound respectively, if the new compound is experimentally characterized using the CiPA dynamic protocol for hERG with manual and CiPA block potency protocols for non-hERG channels with the HTS system. Of note if the validation results support the use of TMS from CiPAORdv1.0 for risk classification, a re-training using all 28 CiPA drugs' data will be performed to

update the two risk categorization thresholds (Threshold 1 and 2) prior to CiPA implementation. Though not the main validation end points, CiPAORdv1.0's ranking or classification performance using the pure HTS dataset can be used to test the possibility of implementing CiPA without any manual voltage clamp data (as might be envisioned in an early drug screening paradigm).

4. Summary

The published CiPAORdv1.0 and qNet metric [1] will be validated with new ionic current data characterizing the electrophysiologic effects of the remaining 16 CiPA drugs. The qNet value averaged across 1x, 2x, 3x, and 4x Cmax will be used as each drug's Torsades Metric Score (TMS). Uncertainty quantification analysis will be performed to estimate the probabilistic distribution of the TMS for each drug. Two separate validation datasets will be used: one pure manual dataset VDMANUAL and one hybrid dataset VDHYBRID that contains manual hERG Milnes data and HTS non-hERG block potency data. For each dataset, the model will be evaluated on its performance on two tasks using TMS: rank order capability, and classification capability. The dataset and performance measure combination leads to four validation endpoints, each corresponding to a unique scenario of implementing the model/metric for decision making.

Fig 2. Relationship between validation endpoints and possible implementation strategy.

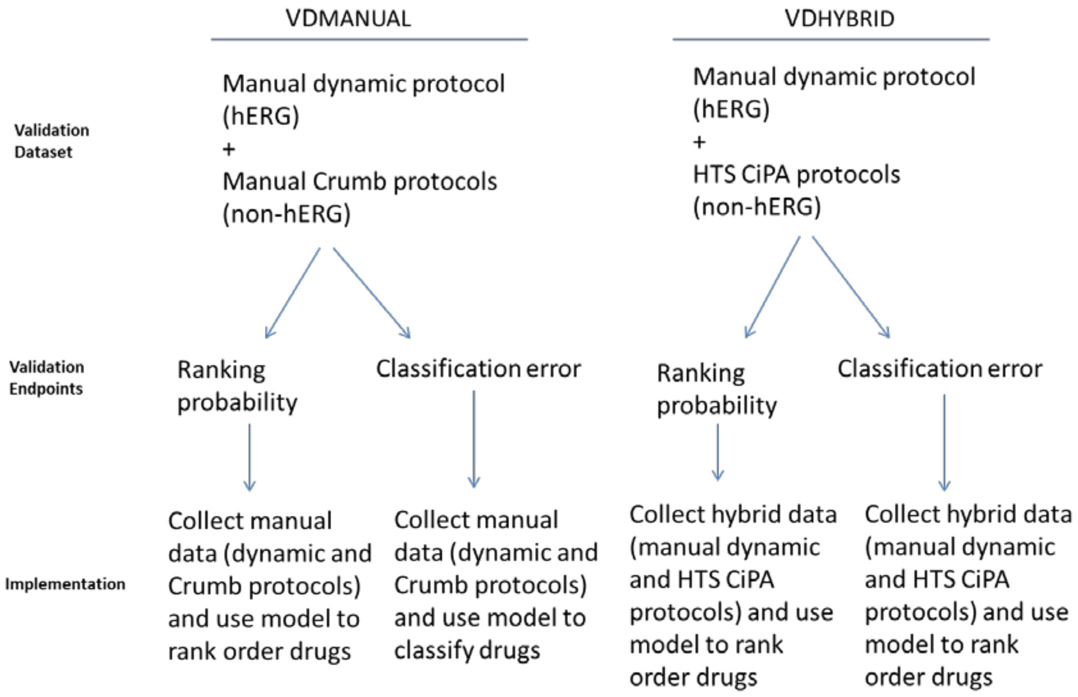

Figure shows various validation endpoints in relation to possible CiPA model implementation strategy. The performance of the four validation endpoints will influence the final implementation of CiPA model in four scenarios as shown on the bottom.

Table 1. Summary of performance measures and pre-defined acceptable performance levels.

Performance Measure	Interpretation	Minimally acceptable performance	Good performance	Excellent performance
AUC of ROC1	Probability of ranking an Intermediate-or-High risk drug above (TMS lower than) a Low risk drug	>~0.7	>~0.8	>~0.9
AUC of ROC2	Probability of ranking a High risk drug above (TMS lower than) an Intermediate-or-Low drug	>~0.7	>~0.8	>~0.9
Pairwise comparison	Probability of correctly ranking a drug relative to CiPA reference drugs through pairwise comparison	>~0.7	>~0.8	>~0.9
LR+ of Threshold 1	Likelihood ratio of a positive prediction (High-or-Intermediate) occurring in High-or-Intermediate risk drugs versus in Low risk drugs	>~2	>~5	>~10
LR- of Threshold 1	Likelihood ratio of a negative prediction (Low risk) occurring in High-or-Intermediate risk drugs versus in Low risk drugs	<~0.5	<~0.2	<~0.1
LR+ of Threshold 2	Likelihood ratio of a positive prediction (High risk) occurring in	>~2	>~5	>~10

	High risk drugs versus in Intermediate-or-Low risk drugs			
LR- of Threshold 2	Likelihood ratio of a negative prediction (Intermediate-or-Low) occurring in High risk drugs versus in Intermediate-or-Low risk drugs	<~0.5	<~0.2	<~0.1
Mean Classification Error	Average error of classifying each of the 16 validation drugs into High, Intermediate, or Low risk category	<~1	<~0.5	<~0.3

For each of the proposed validation measures, three levels of acceptable performance values are defined: minimally acceptable, good, and excellent. These levels were defined according to the current practice and thinking of evaluating diagnostic models or tests. See main text for the references and rationale for these values.

References

1. Dutta, S., et al., *Optimization of an In silico Cardiac Cell Model for Proarrhythmia Risk Assessment.* Front Physiol, 2017. **8**: p. 616.
2. Li, Z., et al., *Improving the In Silico Assessment of Proarrhythmia Risk by Combining hERG (Human Ether-a-go-go-Related Gene) Channel-Drug Binding Kinetics and Multichannel Pharmacology.* Circ Arrhythm Electrophysiol, 2017. **10**(2): p. e004628.
3. Crumb, W.J., Jr., et al., *An evaluation of 30 clinical drugs against the comprehensive in vitro proarrhythmia assay (CiPA) proposed ion channel panel.* J Pharmacol Toxicol Methods, 2016.
4. Chang, K.C., et al., *Uncertainty Quantification Reveals the Importance of Data Variability and Experimental Design Considerations for in Silico Proarrhythmia Risk Assessment.* Frontiers in Physiology, 2017. **8**(917).

5. Taylor, J.M., D.P. Ankerst, and R.R. Andridge, *Validation of biomarker-based risk prediction models.* Clin Cancer Res, 2008. **14**(19): p. 5977-83.
6. Steyerberg, E.W., et al., *Assessing the performance of prediction models: a framework for traditional and novel measures.* Epidemiology, 2010. **21**(1): p. 128-38.
7. Grund, B. and C. Sabin, *Analysis of biomarker data: logs, odds ratios, and receiver operating characteristic curves.* Curr Opin HIV AIDS, 2010. **5**(6): p. 473-9.
8. Mossman, D., *Three-way ROCs.* Med Decis Making, 1999. **19**(1): p. 78-89.
9. Simundic, A.M., *Measures of Diagnostic Accuracy: Basic Definitions.* EJIFCC, 2009. **19**(4): p. 203-11.
10. Kramer, J., et al., *MICE models: superior to the HERG model in predicting Torsade de Pointes.* Sci Rep, 2013. **3**: p. 2100.
11. Lancaster, M.C. and E.A. Sobie, *Improved Prediction of Drug-Induced Torsades de Pointes Through Simulations of Dynamics and Machine Learning Algorithms.* Clin Pharmacol Ther, 2016. **100**(4): p. 371-9.
12. Nooraee, N., G. Molenberghs, and E.R. van den Heuvel, *GEE for longitudinal ordinal data: Comparing R-geepack, R-multgee, R-repolr, SAS-GENMOD, SPSS-GENLIN.* Computational Statistics and Data Analysis, 2014. **77**: p. 70-83.
13. Parsons, N.R., et al., *Repeated measures proportional odds logistic regression analysis of ordinal score data in the statistical software package R.* Computational Statistics and Data Analysis, 2009. **53**: p. 632-641.
14. Touloumis, A., A. Agresti, and M. Kateri, *GEE for multinomial responses using a local odds ratios parameterization.* Biometrics, 2013. **69**(3): p. 633-40.
15. Guyatt, G., et al., *User's Guides to the Medical Literature: A Manual for Evidence-Based Clinical Practice (2nd Edition)*. 2002: McGraw-Hill Professional.
16. Sager, P.T., et al., *Rechanneling the cardiac proarrhythmia safety paradigm: a meeting report from the Cardiac Safety Research Consortium.* Am Heart J, 2014. **167**(3): p. 292-300.
17. Mirams, G.R., et al., *Simulation of multiple ion channel block provides improved early prediction of compounds' clinical torsadogenic risk.* Cardiovasc Res, 2011. **91**(1): p. 53-61.

Signatures Page

Prepared by

Zhihua Li -S

Digitally signed by Zhihua Li -S
DN: c=US, o=U.S. Government, ou=HHS, ou=FDA, ou=People, cn=Zhihua Li -S, 0.9.2342.19200300.100.1.1=2000931442
Date: 2017.11.27 14:27:41 -05'00'

Nov 27th, 2017

Zhihua Li, PhD — Date

Interdisciplinary Scientist, Division of Applied Regulatory

Science, U.S. Food and Drug Administration

Approved by

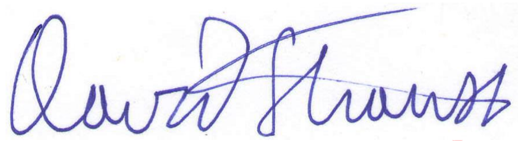

Digitally signed by David Strauss -S
DN: c=US, o=U.S. Government, ou=HHS, ou=FDA, ou=People, cn=David Strauss -S, 0.9.2342.19200300.100.1.1=2000507494
Date: 2017.11.27 14:58:36 -05'00'

David Strauss, MD, PhD — Date

Director, Division of Applied Regulatory Science

U.S. Food and Drug Administration
