# Supplementary material for: Assessment of an In Silico Mechanistic Model for Proarrhythmia Risk Prediction Under the CiPA Initiative
Source: Clin Pharmacol Ther. 2018 Aug 27;105(2):466–75. doi: 10.1002/cpt.1184 (PMC6492074; doi:10.1002/cpt.1184)
Supplement: Supplementary file 2 — Supplementary Text S2. CiPA in silico model validation report. [file CPT-105-466-s002.pdf]

CiPA *In Silico* Model Validation Report

CiPA *In Silico* Working Group

6/25/2018

1. Background

Under the Comprehensive In Vitro Proarrhythmia Assay (CiPA) Initiative, the candidate model (CiPAORdv1.0) (1) and metric (qNet/Torsade Metric Score) established by training data have been used to predict the TdP risk levels of the 16 validation drugs and the prediction performance is assessed according to the CiPA In Silico Model Validation Strategy (hereinafter referred to as Validation Strategy) prespecified prior to validation. This report contains the technical details and comprehensive results for this validation study.

2. Model, Metric, and Validation Datasets

The model (CiPAORdv1.0) and metric (qNet/Torsade Metric Score) were all “frozen” in previous publications based on training data (1-3), and documented in Validation Strategy prior to validation. Similar to training data, two semi-independent validation datasets, one manual and one hybrid, were generated for the same set of 16 CiPA drugs. The two thresholds that classify drugs into three TdP risk categories were also calculated (see section 6 of this document for calculation method) and “frozen” based on the training data. For the manual dataset, Threshold 1 (separating Low from Intermediate/High Risk) has a value of 0.0689 and Threshold 2 (separating High from Intermediate/Low Risk) has a value of 0.0579 µC/µF. For the hybrid dataset, the two thresholds were calculated as 0.0671 and 0.0581 µC/µF respectively.

The different experimental conditions for manual and hybrid datasets are listed below in Table 1. The detailed experimental procedures used to generate these two datasets are provided in section 3 of this document.

Table 1

	Manual Dataset	Hybrid Dataset
IKr	Protocol: hERG dynamic (Milnes)<br>System: Manual<br>Temperature: Physiological	(using manual data)
ICaL	Protocol: Action Potential (AP)<br>System: Manual; Ba^{2+} as charge carrier<br>Temperature: Physiological	Protocol: ICWG protocol 1<br>System: HTS; Ca^{2+} as charge carrier<br>Temperature: Ambient
INaL	Protocol: ICWG protocol 2<br>System: Manual<br>(veratridine as INaL enhancer)<br>Temperature: Physiological	Protocol: ICWG protocol 2<br>System: HTS<br>(ATXII as INaL enhancer)<br>Temperature: Ambient

INa	Protocol: ICWG protocol 2<br><br>System: Manual<br><br>Temperature: Physiological	Protocol: ICWG protocol 2<br><br>System: HTS<br><br>Temperature: Ambient

A comparison of experimental conditions (voltage protocols, patch clamp systems, etc.) between the manual and the hybrid dataset. ICWG: Ion Channel Working Group of the CiPA Initiative. HTS: High Throughput automated patch clamp System. The details of all voltage protocols and experimental configuration can be found in different parts of Experimental Procedures below. The manual and hybrid dataset share the same data source for IKr/hERG.

3. Experimental Procedures

<u>Manual Patch Clamp Experiments to Assess Drug Binding Dynamics on hERG channel</u>

The experimental procedures were consistent with what were used for to acquire training drug data earlier (3). Experiments were performed on a HEK293 cell line that stably expresses hERG1a subunit (provided by Dr. Gail Robertson, University of Wisconsin-Madison) (4). Cells were passaged using trypsin, and seeded onto small sterilized glass coverslips in 35 mm petri dishes containing minimum essential media (MEM; Gibco, Thermo Fisher Scientific) supplemented with 10% fetal bovine serum (Gibco,Thermo Fisher Scientific) and geneticin (G418; 100 μg/mL; Gibco, Thermo Fisher Scientific). Following passage, cells were incubated at 37°C for a minimum of 24 hrs prior to use for electrophysiology studies. All cells were studied within 56 hrs of seeding. The following drugs and solvent were purchased from Sigma-Aldrich: azimilide dihydrochloride (SML0353), clarithromycin (A3487), disopyramide (D2920000),

tamoxifen (T5648), metoprolo (1441301), and DMSO (D8418). Drugs purchased from Tocris Bioscience include astemizole (3489), clozapine (0444), droperidol (5858), ibultilide (3908), loratadine (1944), nifedipine (1075), nitrendipine (0601), pimozide (0937), risperidone (2865), and E-4031 dihydrochloride (1808). Vandetanib was purchased from Advanced ChemBlocks Inc (10403). To make stock solutions, disopyramide, E-4031, metoprolol, and tamoxifen were dissolved in water. Azimilide, dihydrochloride, clarithromycin, astemizole, clozapine, droperidol, ibultilide, loratadine, nifedipine, nitrendipine, pimozide, and risperidone were dissolved in DMSO. When DMSO was used as a solvent to prepare stock solution, the final DMSO concentration applied to cells did not exceed 0.1%.

All patch clamp experiments were conducted at 37°C. Glass coverslips with cells were placed in a recording chamber mounted on an inverted (Zeiss Axiovert 135TV) or an upright microscope (Zeiss AxioExaminer D1), and the recording chamber was continuously perfused with an external solution flowing at a rate of 2-3 mL/min and containing the following (in mM): 130 NaCl, 10 HEPES, 5 KCl, 1 $MgCl_2$*6H_2O, 1 $CaCl_2$*H_2O, 12.5 dextrose; pH adjusted to 7.4 with 5 M NaOH; ~280 mOsM. Cells were visualized using phase contrast method for the inverted microscope and differential interference contrast-infrared method for the upright microscope for guided patching. Recording electrodes were pulled from filamented borosilicate glass pipettes (BF150-86-10; Sutter Instrument, CA), and had tip resistances < 3 MΩ when filled with the following internal solution (in mM): 120 K-gluconate, 20 KCl, 10 HEPES, 5 EGTA, 1.5 MgATP; pH adjusted to 7.3 with 1 M KOH; ~280 mOsM. Temperatures of the in-line solution heater and recording chamber were maintained with a dual channel temperature controller (TC2BIP from Cell MicroControls for the inverted microscope setup; TC-344C from Warner Instruments for the upright microscope setup), and temperature of the perfusate near the cells was recorded throughout the experiment with an additional thermistor placed inside the recording chamber near the recording site. Recordings were obtained using a Multiclamp 700B amplifier (Molecular

Devices, CA). For whole-cell voltage clamp recordings, the command potential values were corrected for the 15 mV liquid junction potential that resulted from using the above internal solution. For protocols involving step voltage waveforms, signals were filtered at 2-3 kHz, digitized using a Digidata 1550A1 interface (Molecular Devices, CA) at 5-10 kHz, and transferred to a computer using pClamp10 software (Molecular Devices, CA). Seal resistance ranged from 2-10 GΩ, and series resistance was electronically compensated for at 80%. HERG current was evoked with a 10 s depolarizing voltage step from -80 mV to 0 mV for 10 s every 25 s. To evaluate the effects of drugs on hERG current, baseline hERG current stability was achieved prior to drug application for every cell. Each cell was exposed only to one concentration of drug.

Manual Patch Clamp Experiments to Collect Drug Block Potency Data on non-hERG Currents (INaL, INa, and ICaL)

Cell lines: The cloned equivalent of the INa (Nav1.5-HEK; SCN5A) and the L-type ICa (hCav1.2/β2/α2δ1-CHO; CACNA1C/ CACNB2/ CACNA2D1) were used in this study. Cells were obtained from CytoBioscience Inc (San Antonio, TX). Cells were maintained in liquid nitrogen as ready to use vials. Each experimental day, a new vial of cells was thawed and used.

Recording solutions: The external solution for Cav1.2 and Nav1.5 currents had a composition of in mM: NaCl 137, KCl 4, $MgCl_2$ 1, CaCl2 1.8, HEPES 10, dextrose 11 with a pH of 7.4, adjusted using NaOH. The internal solution was (in mM): CsCl 130, $MgCl_2$ 1, NaCl 7, HEPES 5, EGTA 5 with a pH of 7.2 adjusted using (CsOH. In Cav1.2 experiments, Ba^{2+} was used as a charge carrier, and 1.8 mM CaCl2 in the external solution was replaced with 4mM $BaCl_2$. Chemicals were obtained from either Sigma-Aldrich (MA) or Fisher Scientific (PA). For INaL

experiments, 50 μM veratridine (Sigma) was added to the external solution to induce the current.

Drugs: Test compounds were obtained from either Tocris Biosciences (MN) (dofetilide, quinidine, ranolazine, verapamil, mexiletine, diltiazem) or Sigma-Aldrich (MA) (all others).

Data acquisition and analysis: Experiments were performed at 36 ± 1 °C. Currents were measured using the whole-cell patch clamp method as previously described (5). Glass pipettes were pulled from borosilicate glass by a horizontal puller (Sutter Instruments, USA). Pipette tip resistance was approximately 1 to 2 MΩ when filled with internal solutions. Series resistance was compensated electronically by approximately 60-80%. An Axopatch 1-B amplifier (Axon Instruments, Foster City, CA) was used for whole-cell voltage clamping. Creation of voltage clamp pulses and data acquisition was controlled by a computer running pClamp software (ver 9.2 Axon Instruments). The voltage protocols used to record the various currents were those used in Crumb et al. (3). A ventricular action potential waveform, given at a pacing rate of 0.1 Hz, was used to elicit Cav1.2 (kindly provided by Dr. Gail Robertson, University of Wisconsin). The block on INa and INaL were measured using ICWG protocol 2, which consists of a hyperpolarizing pulse to -120 mV for 200 ms, depolarization to -15 mV for a 40 ms, followed by step to 40 mV for 200 ms and finally a 100 ms ramp (1.2 V/s) to a holding potential of -80 mV, repeated every 10sec. INa current was measured as the peak inward current during the step to -15 mV, while INaL current was measured at the end of the -15 mV step.

High Throughput Patch Clamp System to Assess Drug Block Potency Data on non-hERG Currents

Cell Lines*:* Cell lines stably expressing human Cav1.2 (hCav1.2/β2/α2δ1-CHO; CACNA1C/ CACNB2/ CACNA2D1; Charles River Laboratories (Wilmington, MA); Catalog #CT6004) and

Nav1.5 (Nav1.5-CHO; SCN5A; Charles River Laboratories (Wilmington, MA); Catalog #CT6007) ion channels were constructed as described previously (6). The cells were maintained in 100-mm cell culture dishes in Ham's F-12 CHO media supplemented with 10% fetal bovine serum, 100 U/mL of penicillin G sodium, 100 mg/mL of streptomycin sulfate, and the appropriate selection antibiotics.

Prior to experiments, the cells were passed in a medium free of selection antibiotics. Cav1.2 expression was induced with tetracycline 16–24 h before recording. Cell density was 50%–70% confluent at the time of harvest. Cells were harvested by washing twice with 15–20 mL of Hank's Balanced Salt Solution (HBSS) and treatment with Accutase (Innovative Cell Technologies, San Diego, CA) solution for 20 minutes. Detached cells were transferred in a 15-mL conical tube and resuspended with addition of 10 mL of HBSS. Then the cells were pelleted at 500 g for 2 minutes, the supernatant was removed, and the cell pellet was resuspended in 10 mL of HBSS. The cell suspension was centrifuged again at 500 g for 2 minutes and the supernatant removed. Finally, the cell pellet was resuspended in 5mL of HEPES-buffered physiological saline (HB-PS): 137 mM NaCl, 4 mM KCl, 1.8 mM $CaCl_2$, 1 mM $MgCl_2$, 10 mM HEPES, and 10 mM glucose, pH adjusted to 7.4 with NaOH, and osmolarity adjusted to 295 ± 5 mOsm. The final cells dilution was $1x10^6$ cells per mL, approximately.

Chemicals used in a solution preparation were purchased from Sigma-Aldrich (St. Louis, MO) and were of ACS reagent grade purity or higher. Stock solutions of test articles were prepared in dimethyl sulfoxide (DMSO) and stored frozen. Each test article formulation was sonicated (Model 2510/5510; Branson Ultrasonics, Danbury, CT) at ambient room temperature for 20 min to facilitate dissolution. For experiments test article concentrations were prepared fresh daily by diluting stock solutions into extracellular solutions (HB-PS buffer) supplemented with 2 mM $CaCl_2$ (Nav1.5 channel) or with 5 mM $CaCl_2$. (Cav1.2 channel). The final solution composition for Nav1.5 channel was 137 mM NaCl, 4 mM KCl, 3.8 mM $CaCl_2$, 1 mM $MgCl_2$, 10 mM HEPES,

and 10mM glucose, pH adjusted to 7.4 with NaOH. The final solution composition for Cav1.2 channel was 137 mM NaCl, 4 mM KCl, 6.8 mM $CaCl_2$, 1 mM $MgCl_2$, 10 mM HEPES, and 10mM glucose, pH adjusted to 7.4 with NaOH. All test and control solutions contained 0.3% DMSO and 0.01% Kolliphor EL. The test article formulations were prepared in 384-well compound plates using Cyclone automated liquid handling system (Caliper Corp.; Princeton, NJ). The internal HEPES-buffered solution consisted of 90 mM CsF, 50 mM CsCl, 5 mM $MgCl_2$, 2.5 mM EGTA, and 10 mM HEPES, pH 7.2 adjusted with CsOH. A stock solution of Escin (perforating agent) was prepared in DMSO (14 mg/mL) and added to the internal solution at a final concentration of 14 mg/mL.

Recordings were performed on IonWorks Barracuda™ system (Molecular Devices; San Jose, CA) in Population Patch-Clamp™ (PPC) mode as previously described (7). The extracellular solution was loaded into the PPC plate wells (11 mL/well) and a cell suspension was added into the wells (9 mL/well). After establishment of a whole-cell configuration (7-min perforation), membrane currents were recorded by IWB on-board patch clamp amplifiers.

Test Article Administration: Test article concentrations were applied to naïve cells (4 wells/ concentration; one concentration per well). Each application consisted of addition of 20 mL of 2X concentrated test article solution to the total 40 mL of final volume of the extracellular well of the PPC planar electrode. Duration of exposure to each test article concentration was five (5) minutes.

hCav1.2 Test Procedures (ICWG Protocol 1): Onset and steady state block of hCav1.2 current was measured using a pulse pattern (ICWG protocol 1), repeated every 10 sec, consisting of a depolarization to 0 mV amplitude for a 40 ms duration, followed by a step to 30 mV for 200 ms and finally a 100 ms ramp (1.2 V/s) to the holding potential of -80 mV. Peak current was measured during the step to 0 mV.

hNav1.5 Peak and Late Current Test Procedures (ICWG Protocol 2): Onset and steady state block of Nav1.5 current was measured using a pulse pattern (ICWG protocol 2), repeated every 5 sec, consisting of a hyperpolarizing pulse to -120 mV for a 200 ms duration, depolarization to -15 mV amplitude for a 40 ms duration, followed by step to 40 mV for 200 ms and, finally, a 100 ms ramp (1.2 V/s) to a holding potential of -80 mV. The peak current was measured during the step to -15 mV. The late Nav1.5 current was measured using the same voltage protocol and all external solutions contained 30 nM ATX-II in order to activate the late current. The Nav1.5 late current was measured at 0 mV potential during the ramp.

Data Analysis: All data were uniformly corrected for run-down:

%Block' = 100%- ((%Block - %PC)*(100% / (%VC - %PC)),

where %VC and %PC are the mean values of the current inhibition with the vehicle and positive controls (highest concentration), respectively.

Acceptance Criteria: Individual well data were filtered according to electrical criteria and the experiments were accepted based on plate level acceptance criteria.

Well Acceptance Criteria:

- Seal Resistance (baseline): $R_{seal} \geq 500$ MW;
- Leak current ≤ 25% peak current;
- Current amplitude (baseline): inward peak current ≥ 0.2 nA

Plate Acceptance Criteria:

- Z' factor (assay sensitivity) ≥ 0.5.

Z' factor for each experiment was calculated as:

Z’ = 1 – ((3x SDVC + 3x SDPC)/ABS (MeanVC – MeanPC),

Where MeanVC and SDVC were the Mean and Standard Deviation values for a vehicle control, MeanPC and SDPC were the Mean and Standard Deviation values for a positive control (1 mM Nifedipine for Cav1.2 and 3 mM Lidocaine for Nav1.5).

4. Uncertainty Quantification of Experimental Data

The manual hERG dynamic data were preprocessed as previously (3) to generate ~1000 points per depolarization sweep (holding at 0 mV for 10 seconds) for each cell. These time course data were used to estimate the 5 drug binding parameters for the dynamic hERG model (3) using the R package deSolve (8) for numerical solving and package cmaes (https://cran.r-project.org/web/packages/cmaes/index.html) for fitting. The cmaes package implements the Covariance Matrix Adaptation Evolution Strategy, and for optimal fitting using averaged time course data we used a population size of 80 and maximum number of generations set to 4000. To quantify the uncertainty in the manual hERG dynamic voltage clamp data, bootstrap was used to generate 2000 samples describing the joint probability distribution of the 5 dynamic parameters for each drug (1) with the R package boot (https://cran.r-project.org/package=boot). Due to the computationally intensive work load a relative tolerance of 10^{-3} was used for numerical integration (deSolve) and a maximum of 100 generations of iterated fitting was used for fitting (cmaes) to the bootstrap samples. The optimal parameter set estimated by averaged time course data across different cells within the same concentration was used as initial parameter guesses for all 2000 bootstrap samples.

For non-hERG (INaL, INa, and ICaL) data from either manual or HTS systems, dose-response (block% at each concentration) data were fitted by a Hill equation to estimate IC50 (half maximal inhibition concentration) and h (Hill coefficient), similar to the training stage (1). To quantify the uncertainty in the dose-response block data, an R package (FME) (9) implementing Markov-chain Monte Carlo (MCMC) simulation was used to generate 2000 MCMC samples describing the joint probability distribution of the Hill equation parameters (IC50 and h).

The above procedures generated 2000 samples (values) for each parameter (binding dynamic parameters for hERG and Hill equation parameters for non-hERG currents) to describe its marginal probability distribution. The 95% credible intervals (CIs, the 2.5-97.5% quantiles of the distributions) for non-hERG parameters of training drugs, hERG parameters of validation drugs, and non-hERG parameters of validation drugs are listed in Tables 2, 3, and 4 respectively. The corresponding values for hERG parameters of training drugs were previously published (1).

Table 2. Block potency (IC50s and Hill coefficients) for training drugs.

Drug channel	IC50 (Manual)	Hill (Manual)	IC50 (Hybrid)	Hill (Hybrid)
**dofetilide INaL**	126 (0.015, 5.68e+06)	1.1 (0.15, 9.5)	837 (0.201, 6.41e+06)	4.6 (0.24, 9.7)
**bepridil INaL**	1.82 (1.56, 2.12)	1.4 (1.1, 1.8)	0.339 (0.3 , 0.415)	1.9 (1.3, 6.5)
**sotalol INaL**	3.28e+03 (2.61e+03, 6.46e+03)	4.8 (1.9, 9.6)	1.34e+05 (810 , 8.03e+06)	5.9 (1.2, 9.8)
**quinidine INaL**	9.46 (7.8 , 12.4)	1.3 (1 , 1.7)	2.36 (1.74, 3.17)	0.91 (0.75, 1.1)
**cisapride INaL**	9.26e+03 (3.43, 7.28e+06)	6.3 (1.8, 9.8)	0.421 (0.324, 0.672)	2.2 (1.1, 9.1)
**terfenadine INaL**	14.9 (1.48, 2.31e+03)	0.66 (0.27, 3.2)	0.0983 (0.0802, 0.124)	1.1 (0.89, 1.5)
**ondansetron INaL**	19.3 (15.8, 24.7)	1 (0.8, 1.4)	6.87 (5.77, 8.27)	1.2 (0.99, 1.6)
**chlorpromazine INaL**	4.59 (3.77, 5.57)	0.94 (0.78, 1.1)	0.673 (0.58, 0.777)	1.8 (1.4, 2.3)
**verapamil INaL**	24.1 (1.32, 3.44e+06)	2 (0.22, 9.4)	0.982 (0.759, 1.44)	1.2 (0.84, 1.8)
**ranolazine INaL**	7.94 (6.2 , 10.3)	0.95 (0.72, 1.3)	5.95 (5.28, 6.64)	0.99 (0.88, 1.1)
**mexiletine INaL**	9.02 (7.71, 11.3)	1.4 (1 , 1.8)	4.69 (3.8 , 5.82)	0.99 (0.85, 1.2)

**diltiazem INaL**	21.6 (16.7, 30.8)	0.68 (0.55, 0.91)	3.04 (2.67, 3.43)	1.1 (0.93, 1.3)
**dofetilide ICaL**	44.5 (0.0123, 5.58e+06)	3.6 (0.32, 9.6)	2.3e+03 (0.329, 6.63e+06)	5.4 (0.8, 9.8)
**bepridil ICaL**	2.82 (1.95, 5.31)	0.65 (0.44, 0.92)	638 (0.455, 6.29e+06)	4.6 (0.29, 9.7)
**sotalol ICaL**	7.13e+03 (4.34e+03, 1.62e+04)	0.87 (0.59, 1.3)	5.8e+04 (588 , 7.83e+06)	5.5 (0.7, 9.8)
**quinidine ICaL**	53.5 (29.6, 141)	0.58 (0.44, 0.74)	5.1e+03 (15.1, 6.53e+06)	4.7 (0.41, 9.7)
**cisapride ICaL**	1.03e+03 (0.359, 6.36e+06)	4.8 (0.41, 9.7)	4.05e+03 (0.86, 7.59e+06)	5.6 (0.88, 9.8)
**terfenadine ICaL**	0.704 (0.612, 0.817)	0.66 (0.59, 0.74)	1.22e+03 (0.505, 6.51e+06)	5.2 (0.46, 9.8)
**ondansetron ICaL**	22.7 (16.1, 38.6)	0.76 (0.55, 1)	9.31e+03 (49 , 6.69e+06)	0.2 (0.1, 0.7)
**chlorpromazine ICaL**	8.32 (6.13, 12.3)	0.85 (0.61, 1.2)	6.35 (3.35, 6.66e+05)	2 (0.26, 9.3)
**verapamil ICaL**	0.204 (0.163, 0.25)	1.1 (0.86, 1.4)	11.2 (1.13, 2.53e+06)	0.8 (0.12, 9.1)
**ranolazine ICaL**	900 (35.2, 5.88e+06)	3.9 (0.49, 9.6)	6.54e+03 (116 , 7.2e+06)	3.8 (0.25, 9.6)
**mexiletine ICaL**	38.9 (22.3, 114)	1 (0.65, 1.6)	164 (96.7, 1.04e+03)	0.96 (0.39, 6.4)
**diltiazem ICaL**	0.113 (0.0747, 0.167)	0.72 (0.53, 1)	31.6 (22.5, 43.5)	1.2 (0.81, 2.1)
**dofetilide INa**	1.36 (0.011, 1.74e+06)	1.1 (0.24, 9.1)	1.46e+03 (0.253, 6.31e+06)	5.1 (0.56, 9.7)
**bepridil INa**	2.96 (2.42, 4.03)	1.2 (0.81, 1.7)	1.61e+03 (0.726, 6.42e+06)	5.4 (0.82, 9.7)
**sotalol INa**	1.12e+05 (3.22e+03, 7.85e+06)	0.86 (0.39, 8.4)	4.95e+04 (547 , 7.11e+06)	3.9 (0.43, 9.7)
**quinidine INa**	12.4 (9.06, 21.5)	1.5 (1 , 2.2)	13 (10.7, 23.2)	2.7 (0.97, 9.4)
**cisapride INa**	1.79e+03 (0.277, 5.32e+06)	0.67 (0.23, 9.4)	16.8 (0.438, 4.15e+06)	2.3 (0.27, 9.6)
**terfenadine INa**	1.73 (0.974, 13.9)	2.4 (0.66, 9.2)	1.95e+03 (0.621, 7.28e+06)	5.3 (0.8, 9.8)
**ondansetron INa**	38.5 (22.5, 301)	1.6 (0.5, 8.7)	19 (11.7, 2.36e+06)	3.7 (0.49, 9.6)
**chlorpromazine INa**	4.58 (3.72, 6.01)	2.1 (1.6, 3.1)	21.2 (3.68, 4.11e+06)	2.5 (0.33, 9.5)
**verapamil INa**	2.59e+03 (2.51, 6.35e+06)	3.5 (0.37, 9.7)	2.48e+03 (1.9 , 7.27e+06)	5.1 (0.64, 9.7)
**ranolazine INa**	53.3 (27.4, 538)	1.9 (0.62, 9.1)	83.7 (58.8, 141)	1.1 (0.7, 1.6)
**mexiletine INa**	26.1 (13.4, 9.46e+05)	3.8 (0.4, 9.5)	67.9 (58.2, 82.1)	1.2 (0.96, 1.6)
**diltiazem INa**	36.9 (15 , 385)	1.4 (0.47, 8.7)	19.7 (17.7, 21.9)	1.3 (1.1, 1.5)

The median values and 95% Credible Intervals (CIs) for IC50s and Hill coefficients obtained using the Markov-chain Monte Carlo simulation pipeline we developed (1) are shown for each drug on each currents. Data from the manual and hybrid datasets are shown together for comparison. For clarity, the values are rounded to limited numbers of significant digits. The

actual values used in the model may be slightly different and can be recreated from the online software release (https://github.com/FDA/CiPA). The unit for IC50s is µM. During simulation the IC50s were converted to nM to match the units in hERG dynamic parameters. For some drugs, the parameters have large 95%CIs that can span several orders of magnitude. As we showed before (1), this is because these drugs do not have significant block even at the highest tested concentrations. The same pattern (that a drug does not have significant block on an ion channel in the tested concentrations) also caused some drug/channel pairs to have very large or very small Hill coefficients. Note that for this table does not include hERG/IKr data, because for this channel/current dynamic parameters, but not IC50s, were used and published before (1).

Table 3. Drug-hERG binding dynamic parameters for the 16 validation drugs.

Drug	Kmax	Ku (ms^{-1})	n	$EC50^n$(nM)	Vhalf-trap (mV)
**vandetanib**	36.28 (4.433, 1.561e+06)	0.01974 (0.01287, 0.08739)	0.7126 (0.5355, 1.348)	2223 (274.8, 3.479e+07)	-48.55 (-72.73, -5.105)
**ibutilide**	14.57 (7.907, 1.805e+05)	6.078e-05 (5.089e-05, 7.399e-05)	0.9231 (0.6182, 1.201)	38.63 (18.97, 3.152e+05)	-9.771 (-38.68, -4.898)
**azimilide**	6.543e+05 (14.45, 5.99e+06)	0.00825 (0.003935, 0.0162)	0.6028 (0.5106, 0.7955)	1.413e+07 (540.9, 1.228e+08)	-8.821 (-35.39, -2.183)
**disopyramide**	3.685 (1.581, 4.027e+05)	0.1216 (0.02537, 0.7438)	0.7894 (0.5, 2)	4.473e+04 (212.3, 4.097e+07)	-78.11 (-91.93, -62.97)
**domperidone**	3.339 (1.341, 2.069e+06)	0.0003558 (0.0002437, 0.0006553)	0.7026 (0.5, 1.23)	16.09 (7.202, 1.049e+07)	-65.65 (-76.66, -6.474)
**droperidol**	14.21 (4.34 , 8.854e+06)	0.001256 (0.0008927, 0.001753)	0.578 (0.5, 0.9603)	116.5 (31.25, 6.581e+07)	-78.68 (-86.43, -70.69)
**pimozide**	10.07 (6.81 , 20.78)	4.576e-05 (3.976e-05, 5.219e-05)	0.8714 (0.5785, 2)	5.601 (2.898, 12.47)	-158.5 (-181.2, -92.29)
**clozapine**	7.486 (3.611, 100.6)	0.02989 (0.01368, 0.1567)	1.367 (0.9507, 2)	9.048e+04 (9695 , 1.343e+07)	-8.81 (-24.61, -3.119)

**risperidone**	3.934 (2.336, 9.025e+05)	0.001151 (0.0007141, 0.001609)	1.122 (0.5, 2)	752.8 (63.83, 1.123e+07)	-80.43 (-157.2, -68.69)
**astemizole**	2.42 (1.688, 4.532)	3.306e-05 (2.139e-05, 4.952e-05)	1.449 (0.9532, 2)	4.883 (2.49 , 10.6)	-6.11 (-70.48, -1.001)
**clarithromycin**	92.89 (5.462, 8.657e+05)	0.01216 (0.008948, 0.01603)	0.7867 (0.6485, 1.178)	2.229e+05 (1.055e+04, 7.933e+08)	-102.8 (-185.9, -78.9)
**tamoxifen**	3.9 (2.52 , 21.62)	0.01175 (0.002841, 0.9834)	2 (1.15 , 2)	4.067e+05 (1.322e+04, 1.305e+06)	-2.036 (-68.43, -1)
**metoprolol**	3.179e+04 (4.568, 5.124e+05)	0.8508 (0.2857, 0.9999)	0.811 (0.589, 1.36)	4.223e+07 (4.045e+04, 8.168e+08)	-89.35 (-178 , -72.79)
**loratadine**	3.765e+05 (7.828e+04, 1.724e+06)	0.009642 (0.0001872, 0.9954)	0.8368 (0.6478, 1.072)	4.77e+08 (7.854e+07, 9.542e+08)	-1 (-15.74, -1)
**nitrendipine**	1.713 (1.137, 6.483)	0.9819 (0.4966, 1)	1.923 (1.001, 2)	5.669e+08 (3.77e+05, 1e+09)	-61.58 (-154.3, -1.001)
**nifedipine**	4.748 (0.7176, 1.917e+04)	0.9916 (0.4739, 1)	1.235 (0.6103, 1.771)	9.752e+08 (1.992e+05, 1e+09)	-87.37 (-177.5, -52.35)

The median and 95% Confidence Interval (CI) values of the five hERG dynamic parameters for each drug are listed. The CIs are calculated based on the bootstrapping method we developed for CiPA (1). For some drugs, the parameters Kmax and $EC50^n$ have large 95%CIs that can span several orders of magnitude. As previously shown, this is because these drugs have a near-linear relationship between the concentration and binding rate, and the ratio $Kmax/EC50^n$, but not the individual parameters, are identifiable (1).

Table 4. Block potency (IC50s and Hill coefficients) for validation drugs.

Drug channel	IC50 (Manual)	Hill (Manual)	IC50 (Hybrid)	Hill (Hybrid)
**vandetanib INaL**	3.79e+03 (42.3, 5.78e+06)	0.82 (0.21, 9.3)	4.21 (3.21, 5.8)	2.2 (1.3, 9)
**ibutilide INaL**	287 (15.8, 5.5e+06)	2.4 (0.32, 9.4)	0.82 (0.657, 1.02)	0.96 (0.79, 1.2)
**azimilide INaL**	2.94e+03 (151 , 1.69e+06)	0.47 (0.22, 1.3)	3.69 (3.19, 4.28)	1.4 (1.1, 1.8)
**disopyramide INaL**	377 (340 , 614)	2.1 (1.8, 9.8)	20.2 (14.7, 27.8)	0.73 (0.57, 0.93)
**domperidone INaL**	225 (26.2, 4.23e+06)	2.1 (0.24, 9.3)	0.674 (0.422, 1.08)	0.93 (0.62, 1.5)

**droperidol INaL**	33.8 (20 , 425)	2.8 (0.66, 9.4)	0.533 (0.386, 0.756)	1 (0.78, 1.4)
**pimozide INaL**	1.91 (0.577, 5.21e+05)	1.9 (0.23, 9.1)	5.03 (0.579, 2e+05)	0.59 (0.15, 4.7)
**clozapine INaL**	73.6 (66.1, 92.2)	2 (1 , 9)	2.24 (1.74, 2.91)	1.4 (1.1, 1.8)
**risperidone INaL**	1.14e+04 (12.5, 7.18e+06)	5.8 (0.89, 9.8)	2.93 (2.18, 53.6)	2.7 (0.48, 9.3)
**astemizole INaL**	10.3 (4.08, 1.45e+03)	2.3 (0.47, 9.1)	0.596 (0.368, 0.963)	3.1 (1.9, 9.5)
**clarithromycin INaL**	1.81e+03 (1.36e+03, 4.97e+03)	3 (0.89, 9.4)	173 (114 , 3.07e+03)	2.4 (0.45, 9.4)
**tamoxifen INaL**	3.64e+03 (42.6, 6.19e+06)	4 (0.54, 9.7)	4.31 (2.73, 7.43)	4.8 (2.8, 9.7)
**metoprolol INaL**	630 (501 , 809)	0.66 (0.58, 0.78)	24.5 (17.6, 33.5)	0.77 (0.62, 0.99)
**loratadine INaL**	192 (61.7, 4.75e+05)	2.1 (0.32, 9.6)	0.937 (0.536, 4.84)	2.8 (0.89, 9.5)
**nitrendipine INaL**	70.7 (40.5, 548)	3.2 (0.96, 9.3)	0.358 (0.259, 0.504)	0.9 (0.8, 1.1)
**nifedipine INaL**	45.6 (11.7, 424)	4.5 (1.5, 9.7)	0.793 (0.471, 1.38)	0.81 (0.66, 1.1)
**vandetanib ICaL**	6.06 (4.57, 8.25)	0.72 (0.6, 0.88)	3.36 (3.06, 4.26)	4.9 (2.1, 9.7)
**ibutilide ICaL**	37 (19.6, 62.5)	0.86 (0.64, 1.6)	18.5 (11.1, 8.78e+05)	2.5 (0.36, 9.6)
**azimilide ICaL**	13.2 (8.29, 23.8)	0.71 (0.48, 1)	12.7 (8.56, 15.8)	4.1 (2 , 9.6)
**disopyramide ICaL**	32.9 (22.9, 46.8)	0.69 (0.56, 0.86)	86.6 (67 , 1.67e+04)	1.6 (0.25, 9.1)
**domperidone ICaL**	0.0736 (0.0438, 0.116)	0.49 (0.38, 0.63)	16.9 (9.42, 21.1)	2.2 (0.86, 9.3)
**droperidol ICaL**	3.23 (1.87, 4.53)	1.2 (0.85, 5.6)	7.63 (4.84, 11.4)	0.93 (0.63, 1.4)
**pimozide ICaL**	0.0645 (0.0431, 0.102)	0.45 (0.36, 0.56)	0.437 (0.29, 0.91)	2.2 (0.62, 9.3)
**clozapine ICaL**	5.49 (4.33, 6.85)	0.94 (0.76, 1.2)	5.33 (1.73, 6.77)	4.7 (1.7, 9.7)
**risperidone ICaL**	1.47 (1.17, 1.94)	0.59 (0.5, 0.69)	5.73e+03 (3.67, 6.68e+06)	5.3 (0.56, 9.7)
**astemizole ICaL**	0.553 (0.474, 0.65)	1.2 (1 , 1.5)	1.08 (0.413, 2.3)	5.9 (2.1, 9.8)
**clarithromycin ICaL**	38.1 (26.5, 54.2)	0.88 (0.68, 1.2)	118 (101 , 257)	3 (0.81, 9.3)
**tamoxifen ICaL**	5.72 (4.38, 7.61)	0.76 (0.61, 0.95)	3.61 (2.56, 6.7)	6.7 (3.1, 9.8)
**metoprolol ICaL**	3.28e+03 (2.03e+03, 7.33e+03)	0.54 (0.39, 0.71)	398 (279 , 702)	0.91 (0.54, 1.5)
**loratadine ICaL**	0.703 (0.55, 0.896)	0.56 (0.5, 0.66)	2.77 (0.634, 21.1)	5.7 (1.5, 9.7)
**nitrendipine ICaL**	0.0357 (0.0182, 0.0734)	0.5 (0.36, 0.93)	1.2 (0.226, 2.81)	1 (0.51, 9.3)
**nifedipine ICaL**	0.0114 (0.00809, 0.0155)	0.67 (0.49, 0.87)	0.0345 (0.0201, 0.0628)	0.86 (0.48, 1.5)
**vandetanib INa**	80.9 (36.5, 9.93e+03)	1.9 (0.37, 9.5)	5.84 (3.67, 11.5)	3.3 (2 , 9.2)
**ibutilide INa**	24.1 (12.4, 483)	2.3 (0.56, 9.5)	8.02 (3.49, 41.1)	0.78 (0.33, 6.5)
**azimilide INa**	363 (36.3, 1.26e+04)	0.72 (0.33, 9.5)	18.4 (16.8, 22.1)	3.8 (1.4, 9.6)
**disopyramide INa**	192 (157 , 233)	1.3 (1.1, 1.6)	72.8 (46.7, 185)	1.2 (0.51, 8.6)

**domperidone INa**	41.9 (22.5, 137)	1.5 (0.61, 9.2)	6.93 (2.47, 16.8)	2.6 (1.1, 9.5)
**droperidol INa**	36.6 (20.3, 1.1e+03)	2.5 (0.53, 8.5)	8.22 (5.93, 11.8)	1.1 (0.82, 1.7)
**pimozide INa**	10.2 (1.77, 3.65e+04)	0.46 (0.17, 0.95)	4.11e+03 (1.67, 7.04e+06)	5.5 (0.97, 9.8)
**clozapine INa**	257 (128 , 1.05e+03)	0.59 (0.36, 0.99)	8.21 (7.43, 10.5)	3.4 (1.5, 9.5)
**risperidone INa**	534 (3.3 , 3.78e+06)	0.71 (0.25, 8.6)	9.76e+03 (8.48, 6.46e+06)	5.5 (0.96, 9.8)
**astemizole INa**	5.41 (3.53, 11.2)	0.76 (0.49, 2)	2.43 (1.79, 2.74)	4.8 (2.1, 9.6)
**clarithromycin INa**	1.09e+03 (957 , 1.26e+03)	0.89 (0.79, 1)	835 (122 , 5.2e+06)	4 (0.45, 9.7)
**tamoxifen INa**	84 (24.3, 4.42e+03)	0.82 (0.29, 8.7)	9.59 (8.67, 10.1)	3.6 (1.9, 9.5)
**metoprolol INa**	30.3 (20.3, 44.4)	0.61 (0.48, 0.77)	150 (123 , 181)	1 (0.82, 1.3)
**loratadine INa**	113 (51.8, 1.72e+03)	1.4 (0.37, 9.6)	23.2 (9.77, 30.7)	3.3 (1.1, 9.5)
**nitrendipine INa**	22.4 (13.9, 43.9)	0.58 (0.38, 0.85)	11.1 (4.17, 25.1)	1.4 (0.85, 8.6)
**nifedipine INa**	27.6 (15.8, 33.4)	1.1 (1 , 2)	46.3 (33 , 53.3)	2.5 (0.98, 9.1)

The median values and 95% Credible Intervals (CIs) for IC50s and Hill coefficients obtained using Markov-chain Monte Carlo simulation similar to Table 2, but with validation drugs. For clarity the values are rounded to limited numbers of significant digits. The actual values used in the model may be slightly different and can be recreated from online software release (https://github.com/FDA/CiPA). The unit for IC50s here is µM. During simulation the IC50s were converted to nM to match the units in hERG dynamic parameters. For those drug/channel pairs that show very large confidence intervals or extreme hill coefficients, please see legend to Table 2 for an explanation.

5. TdP Risk Levels and Clinical Exposure of Validation Drugs (Cmax)

The known TdP categories and Cmax (maximum free therapeutic concentration) for validation drugs are listed in **Table 5** below. For droperidol, only the total therapeutic Cmax was reported in the literature with a value of 60 ng/ml (10). No protein binding assays were reported for this drug, but one study reported the human serum albumin association constant (Ka) for a series of psychoactive butyrophenones including droperidol (11). By definition

$$Ka = \frac{[RL]}{[R] * [RL]}$$

$$B = \frac{[RL]}{[RL] + [L]}$$

Where [RL], [R] and [L] are concentrations for bound ligand (bound drug), free receptor (albumin), and free ligand (free drug) respectively, and B represents the protein binding fraction. If the Ka values for two drugs (Ka1 and Ka2) are known, and the B value for one of them (B2) is also known, then the B value for the other drug (B1) can be calculated as

$$B1 = \frac{1}{\frac{Ka2}{Ka1} * \left(\frac{1}{B2} - 1\right) + 1}$$

In that report haloperidol has a Ka of 0.86 1/M and protein binding rate of 93%, while droperidol has a Ka of 1.68 1/M. Using the equation above we calculated protein binding rate for droperidol as 96%. Together with the total Cmax and a molecular weight of 379.4, this gave a free Cmax of 6.33 nM for droperidol.

Table 5. CiPA TdP risk levels and Cmax for the 16 validation drugs.

**Drug**	**TdP Category**	**Cmax (nM)**	**Ref**
**vandetanib**	High	255.4*	FDA label
**ibutilide**	High	140**	Redfern et al.(10)
**azimilide**	High	70	Redfern et al.(10)
**disopyramide**	High	742	Redfern et al.(10)
**domperidone**	Intermediate	19	Redfern et al.(10)
**droperidol**	Intermediate	6.33***	Multiple sources (10, 11)
**pimozide**	Intermediate	0.431	Redfern et al.(10)
**clozapine**	Intermediate	71	Kramer et al.(12)
**risperidone**	Intermediate	1.81	Redfern et al.(10)
**astemizole**	Intermediate	0.26	Redfern et al.(10)
**clarithromycin**	Intermediate	1206	Redfern et al.(10)
**tamoxifen**	Low/No	21	Redfern et al.(10)
**metoprolol**	Low/No	1800****	Multiple sources (13, 14)
**loratadine**	Low/No	0.45	Redfern et al.(10)
**nitrendipine**	Low/No	3.02	Redfern et al.(10)

**nifedipine**	Low/No	7.7	Redfern et al.(10)

The CiPA TdP risk categories as well as free maximum therapeutic concentrations (Cmax) for the 16 validation drugs. The references for the Cmax are also shown.

*Based on a total Cmax of 2024 ng/ml and a plasma protein binding rate of 94%, both from FDA label (https://www.accessdata.fda.gov/drugsatfda_docs/nda/2011/022405Orig1s000ClinPharmR.pdf)

**Due to its extreme potency on hERG, the highest concentration tested on the ibutilide hERG assay was 100 nM. Because the metric calculation is most reliable up to the highest concentrations with experimental data (1), we used 100 nM instead of 140 nM as ibutilide's Cmax in calculating its qNet and *torsade metric score*. This is not projected to change the prediction results.

***The Redfern paper only listed the total Cmax of droperidol as 60 ng/ml. We calculated the (free) Cmax here based on this total Cmax and one study reporting the binding association constant between droperidol and human serum albumin (11). See main text above for details.

****Metoprolol has a maximum total Cmax of 221 nM at a dose of 50 mg (14) and a protein binding rate of 90% (13). According to FDA label (https://www.accessdata.fda.gov/drugsatfda_docs/label/2008/017963s062,018704s021lbl.pdf), the maximum daily dose is 450 mg and there is a linear relationship between oral dose and plasma concentration up to 400 mg. A free Cmax of ~1800 nM was calculated assuming a linear PK profile up to a single oral dose of 450 mg.

6. Modeling Procedures

Human Ventricular Model and TdP Risk Metric

The numerical methods and simulation procedures were published in details previously (1). Briefly, the optimized IKr-dynamic ORd model, which includes the dynamic hERG model as a component (3) and was developed using training data (2), was frozen as CiPAORdv1.0 model for validation in this study. Model equations were written in C and compiled for use with R (https://www.r-project.org) and the deSolve package (8). Equations were integrated using the lsoda solver with relative and absolute error tolerance both set at 10^{-6}.

To calculate the *torsade metric score* (TMS), the CiPAORdv1.0 model was used to simulate action potentials (APs) at a cycle length of 2s (to mimic bradycardia) for 1000 beats, starting from control conditions achieved by pacing without drug for 1000 times to reach steady state (1). For each drug, 4 concentrations (1-4x Cmax) were used to calculate the metrics, which were then averaged across concentrations to obtain a mean metric value for each drug. For qNet, this mean value is *torsade metric score* (TMS). Other metrics (like APD90) were calculated in a similar way, averaging across 1-4x Cmax.

The qNet metric (2) was computed by integrating the sum of six major currents (IKr, ICaL, INaL, Ito, IKs, and IK1) over one of the last 250 beats with the steepest reactivation of the membrane potential during the plateau phase (as this is the “worst case scenario” in terms of EAD generation) (1). APD90 and APD50 were defined as the time it takes an AP to repolarize by 90% and 50% respectively, between the peak of AP and the resting membrane potential. When APD90 or APD50 were included in the metric some drugs may have undeterminable metric value as there are repolarization failures at the concentrations tested. Those cases were assigned to High Risk category automatically. Note though this only applies to those metrics that are dependent on AP wave forms. For qNet/TMS, the metric values can still be calculated

even in the presence of repolarization failure. Diastolic Ca^{2+} concentration was calculated as the minimum Ca^{2+} during the resting membrane potential stage.

For uncertainty propagation, the bootstrap samples from drug-hERG dynamics and MCMC samples from non-hERG block potency for each drug from uncertainty quantification (section 4 of this document) above were assumed to be independent and combined sequentially to form 2000 drug-effect samples, which were then fed into the CiPAORdv1.0 to calculate 2000 qNet values for each drug at each concentration. The qNet values from 1 to 4x Cmax were averaged to define a TMS distributions with 2000 sample values for each drug to predict the TdP risk.

Calculating Classification Thresholds from Training Data

To classify drugs into distinct TdP risk categories, a classifier was trained on all TMS distributions of all 12 drugs using ordinal logistic regression implemented in the R package rms (https://CRAN.R-project.org/package=rms). For the consideration of inter-correlation between all samples for the same drug please refer to the prespecified **Validation Strategy**.

The classifier reports the probability of each sample belonging to each of the three risk categories, and the sample was assigned to the category with the highest probability. To convert this process into classification thresholds for visualization, the follow procedure was taken.

The ordinal logistic regression can be written as

$$P(risk \leq Low) = \frac{1}{1 + e^{-(I1+\ \beta * TMS)}}$$

$$P(risk \leq Intermediate) = \frac{1}{1 + e^{-(I2+\ \beta * TMS)}}$$

Where *I1* and *I2* are the intercepts, and β the coefficient, of the linear equation that maps TMS, through a logistic link function, to the cumulative probabilities of being in a risk category. Threshold 1 (separating Low from Intermediate/High Risk) is defined as the TMS value where the probability of being in the Low category is equal to that in the Intermediate category, so when TMS=Threshold 1

$$P(risk \leq Low) = 0.5 * P(risk \leq Intermediate)$$

And the corresponding Threshold 1 can be calculated as

$$\text{Threshold 1} = \log(e^{-I2} - 2 * e^{-I1})/\beta$$

Threshold 2 (separating High from Low/Intermediate Risk) is defined as the TMS value where the probability of being in the High category is equal to that in the Intermediate category, so when TMS=Threshold 2

$$1 - P(risk \leq Intermediate) = P(risk \leq Intermediate) - P(risk \leq Low)$$

And the corresponding Threshold 2 can be calculated as

$$\text{Threshold 2} = \log(e^{-I1} * e^{-I2}/-(2 * e^{-I1} - e^{-I2}))\,/\beta$$

After this calculation, a drug sample with its TMS right to (greater than) Threshold 1 would have its highest probability in the Low Risk category, while a drug sample whose TMS is left to (less than) Threshold 2 would have its highest probability in the High Risk category. A drug sample whose TMS is in between Threshold 1 and 2 would have the highest probability in the Intermediate Risk category.

7. Performance Measures

The model performance measures on the validation drugs were pre-specified in **Validation Strategy**, where the rationale and analytical methods for these measures are also provided. Briefly, Samples from the probability distributions of TMS for each drug were randomly sampled to form 10000 datasets, each containing 16 metric values for 16 drugs. Performance measure was calculated on each of the 10000 datasets, and measures across all datasets were used to estimate the median and 95% confidence interval (CI) values, where 95% CI was calculated as the 2.5% - 97.5% percentile of the measurements across 10000 datasets.

Receiver Operating Characteristic as a Rank Performance Measure

The Receiver Operating Characteristic (ROC) curve was constructed by taking all possible TMS cutoffs (thresholds) for the 16 validation drugs to plot sensitivity vs 1-specificity. For ROC1 sensitivity is the proportion of true High-or-Intermediate Risk drugs predicted to be so, while specificity is the proportion of true Low Risk drugs predicted to be so. For ROC2 sensitivity is the proportion of true High Risk drugs predicted to be so, while specificity is the proportion of true Intermediate-or-Low Risk drugs predicted to be so. Area under the curve (AUC) of ROC was calculated by the R package ROCR (15). The representative ROC curves as well as distribution of AUCs across 10000 curves for ROC1 analysis are shown for the manual (**Fig 1A**) and hybrid (**Fig 1B**) validation dataset respectively. The results suggest that the probability of ranking a High-or-Intermediate Risk drug above (*torsade metric score* lower than) a Low Risk drug using CiPAORdv1.0 is 0.89 (95%CI 0.84 - 0.95) and 0.98 (0.93 - 1) for manual and hybrid validation dataset, respectively. Similarly, for ROC2 analysis (**Fig 2A&B**), the probability of ranking a High Risk drug above (*torsade metric score* lower than) an Intermediate-or-Low Risk drug is 1 (0.92 - 1) and 0.94 (0.88 - 0.98) for manual and hybrid validation dataset, respectively.

The median values of all these AUCs exceed or are very close to the pre-defined "excellent" ranking performance level (AUC > ~0.9) (Table 1 of **Validation Strategy**).

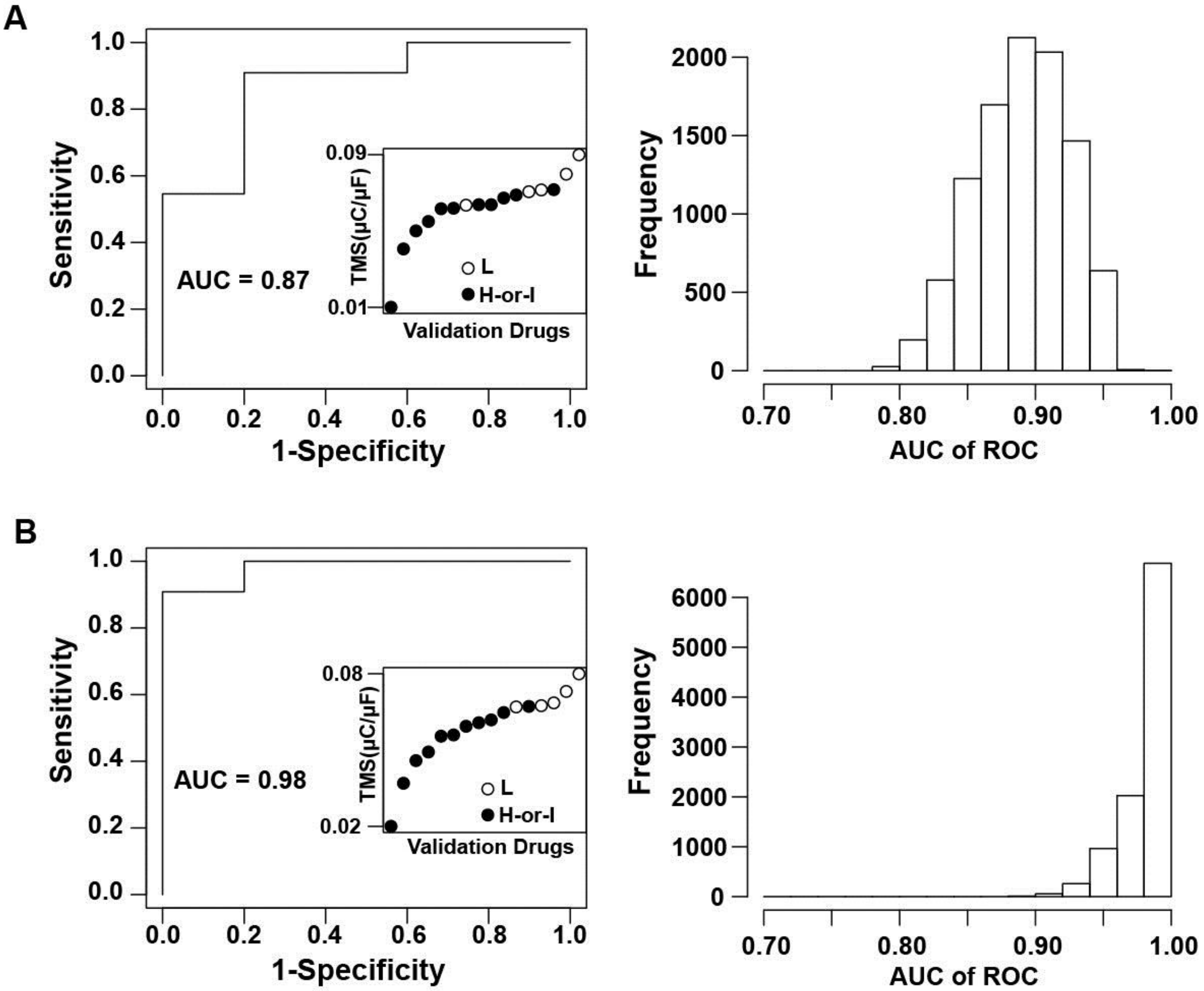

Figure 1. ROC1 Analysis to Estimate the Probability of Ranking High-or-Intermediate Drugs Above Low Risk Drugs.

For ROC1 analysis, High and Intermediate Risk drugs are combined into one category (High-or-Intermediate), and 10000 ROC curves are constructed by sampling the *torsade metric score*

distributions for the manual (**A**) and hybrid (**B**) validation dataset respectively. Left panel of **A** or **B**: one representative example of the 10000 ROC curves and the corresponding AUC. Insert: the underlying ranking of the 16 drugs (X axis: rank of 1-16; drug names not shown on X axis for figure clarity) for this particular ROC curve according to their *torsade metric score* values (Y axis); L: Low/No Risk Drugs; H-or-I: High-or-Intermediate Risk Drugs. Note that H-or-I drugs (black) generally have a *torsade metric score* lower than L drugs (white), indicating a higher ranking of TdP risk. Right panel of **A** or **B**: Distribution of the AUCs across the 10000 ROC curves.

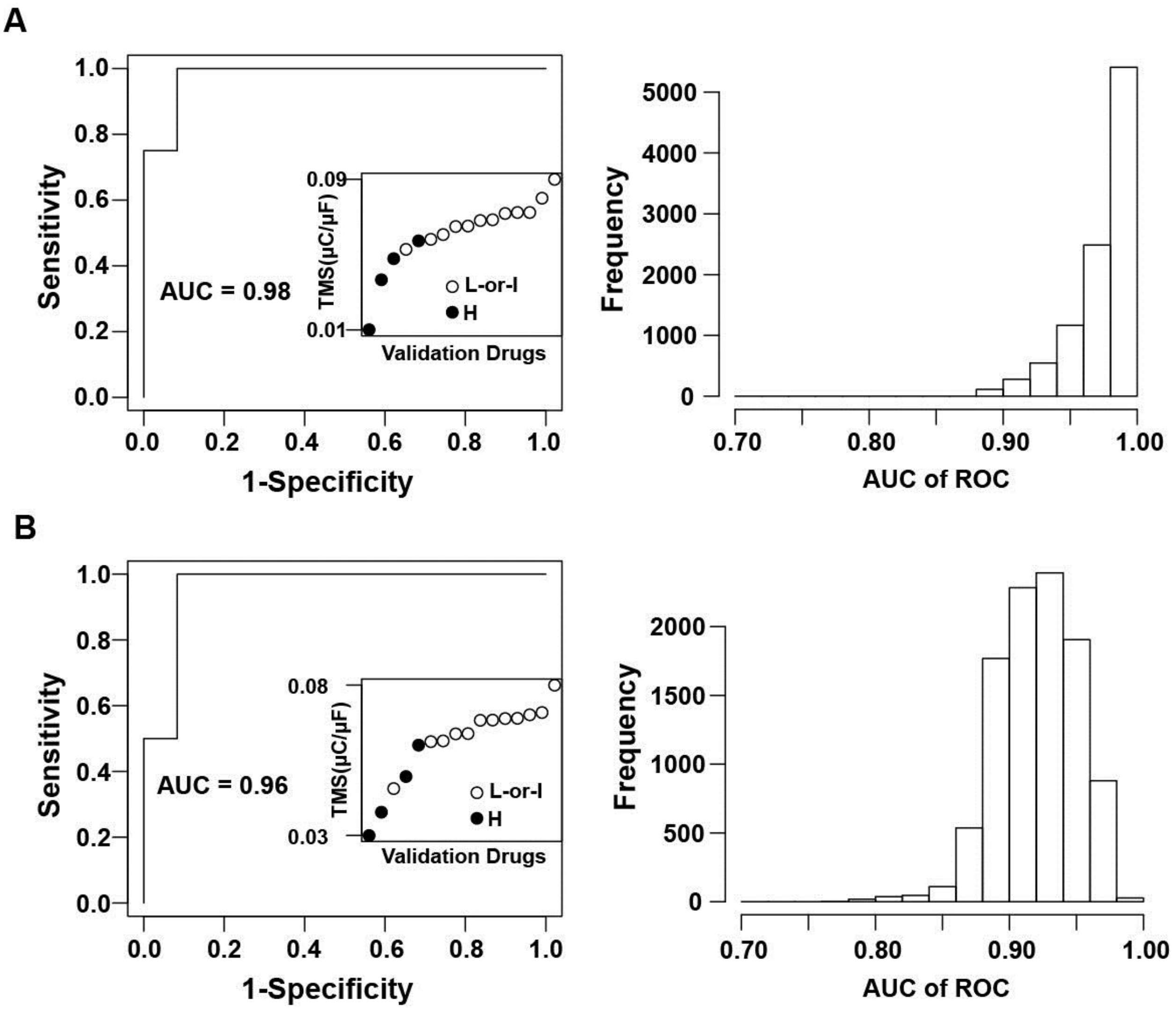

Figure 2. ROC2 Analysis to Estimate the Probability of Ranking High Risk Drugs Above Low-or-Intermediate Risk Drugs.

For ROC2 analysis, Low and Intermediate Risk drugs are combined into one category (Low-or-Intermediate), and 10000 ROC curves are constructed by sampling the *torsade metric score* distributions for the manual (**A**) and hybrid (**B**) validation dataset respectively. Left panel of **A** or **B**: one example of the 10000 ROC curves and the corresponding AUC. Insert: the underlying ranking of the 16 drugs (X axis: rank of 1-16; drug names not shown for figure clarity) for this particular ROC curve according to their *torsade metric score* values (Y axis); L-or-I: Low-or-

Intermediate Risk Drugs; H: High Risk Drugs. Note that H drugs (black) generally have a *torsade metric score* lower than L-or-I drugs (white), indicating a higher ranking of TdP risk. Right panel of **A** or **B**: Distribution of the AUCs across the 10,000 ROC curves.

Pairwise Comparison as a Rank Performance Measure

Out of the 378 (28*27/2) theoretical pairwise combinations of the 28 CiPA drugs, drug pairs where both compounds are from the same category (so the exact ranking is unclear), or both compounds are from the training set (so the information has already been used for model building), were removed, giving rise to 211 validation pairs. The *torsade metric score* distributions for validation drugs were randomly sampled (with replacement) to calculate the percentage of correctly predicted pairs among the 211 pairs 10000 times. A representative example, as well as the distribution of the correct-prediction rate across all 10000 analyses, is shown for the manual (**Fig 3 A**) and hybrid (**Fig3 B**) validation datasets respectively. This analysis indicates that the probability of correctly ranking a drug through pairwise comparison against the CiPA reference drugs is 0.95 (95% CI 0.92 – 0.98) and 0.96 (0.92 – 0.99) for the manual and hybrid dataset respectively, well above the prespecified “excellent” performance level (> ~0.9). In addition, the **Validation Strategy** specifies a measure where each validation drug’s predicted TdP risk rank among all 28 CiPA reference drugs is compared to the true rank. The data are shown in **Table 6**, which show a high level of consistency and low variability (relatively narrow 95%CI).

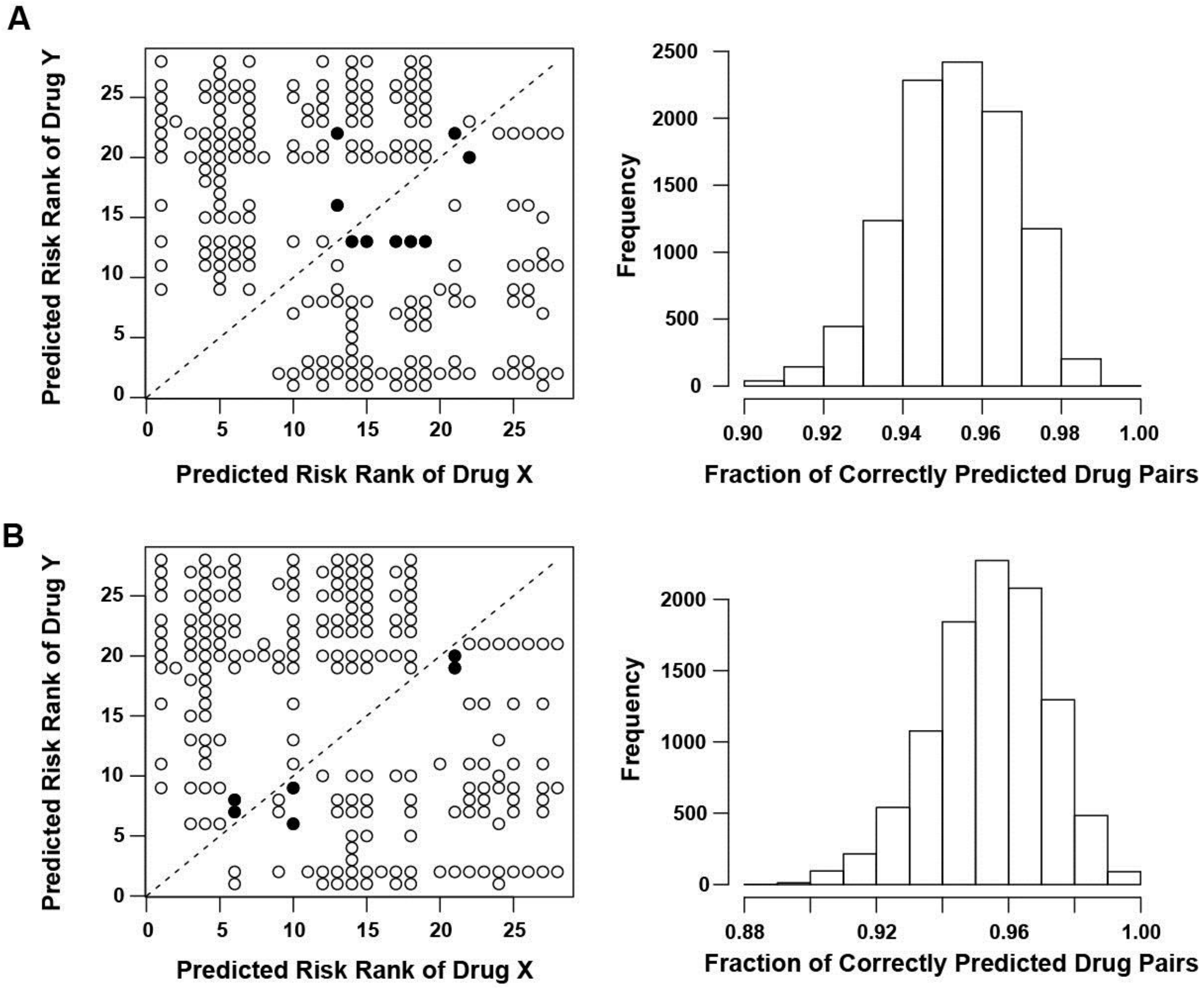

Figure 3. Pairwise Comparison to Estimate the Probability of Correctly Ranking a Drug Across All Three TdP Categories of the CiPA Reference Drugs.

A) Results for the manual validation dataset. Left: one example of the 10000 pairwise comparison analyses, each with 211 drug pairs. Each point represents one of 211 drug pairs, with the X (or Y) axis indicating the predicted ranking of one (or the other) drug in the pair among the 28 CiPA reference drugs. Correctly predicted drug pairs are represented by white points, while incorrectly predicted drug pairs black points. Right: distribution of the fraction of

correctly predicted drug pairs across 10000 analyses. **B**) Similar to A but for the hybrid validation datasets.

Table 6. Known and Predicted TdP Risk Rank for Validation Drugs.

drug	TdP Risk Rank	Predicted Rank (manual)	Predicted Rank (hybrid)
**ibutilide**	1-8	1(1 - 1)	1(1 - 1)
**azimilide**	1-8	5(4 - 6)	4(3 - 7)
**disopyramide**	1-8	8(7 - 13)	12(9 - 18)
**vandetanib**	1-8	3(2 - 3)	2(2 - 2)
**clarithromycin**	9-19	18(16 - 20)	15(12 - 17)
**risperidone**	9-19	21(18 - 22)	20(17 - 22)
**domperidone**	9-19	10(7 - 16)	6(3 - 9)
**astemizole**	9-19	12(8 - 16)	11(8 - 15)
**pimozide**	9-19	14(8 - 17)	9(7 - 14)
**droperidol**	9-19	12(8 - 16)	13(9 - 16)
**clozapine**	9-19	19(17 - 21)	19(16 - 22)
**metoprolol**	20-28	12(8 - 16)	25(20 - 26)
**loratadine**	20-28	22(21 - 23)	22(20 - 23)
**tamoxifen**	20-28	20(19 - 22)	20(18 - 21)
**nifedipine**	20-28	26(26 - 27)	27(27 - 27)
**nitrendipine**	20-28	25(24 - 25)	23(22 - 25)

For each validation drugs, the known CiPA TdP risk rank against all 28 reference drugs are compared to predicted risk rank for the manual and hybrid dataset. The most dangerous drug has a rank of 1, while the safest drug a rank of 28. Note that because with-in CiPA risk category ranking is unknown, each drug has a range of possible risk rank (column 2). High Risk drugs have a range of 1-8, while Intermediate and Low Risk drugs have a range of 9-19 and 20-28 respectively. The median and 95%CI of the predicted ranks for the manual and hybrid validation dataset are shown in columns 3&4. The 95% CI are based on the 10000 subsets sampled from the probability distribution of TMS.

Likelihood Ratio as a Classification Performance Measure

Likelihood Ratio for positive results (LR+) was calculated as sensitivity divided by 1-specificity, while likelihood ratio for negative results (LR-) was 1-sensitivity divided by specificity. To prevent the denominator from becoming 0, a very small number, randomly sampled from a normal distribution ($\mu=1x10^{-6}$ and $\delta=1x10^{-12}$), was added to both the dividend and denominator. In the calculation of LR+ and LR- for Threshold 1, the definition of sensitivity and specificity is the same as in ROC1, while for Threshold 2 the definition is the same as in ROC2. Note though that unlike ROC analysis, where many thresholds were tried to calculate a series of sensitivity and specificity, for LR analysis sensitivity and specificity were based on specific thresholds (Threshold 1&2 as defined in section 2 of this document) pre-defined by training data.

The resulting values (Table 7**)** suggest that, using Threshold 1 as a cutoff value, a High-or-Intermediate Risk drug is 4.5 and $8x10^5$ times (median of LR+) more likely to be classified into the High-or-Intermediate category, but 8.8 and 5.5 times (median of 1/LR-) less likely to be classified into the Low Risk category, compared to a Low Risk drug, for the manual and hybrid validation dataset, respectively. Similarly, using Threshold 2, a High Risk drug is 12 and 6 times more likely to be classified into the High category, but $9x10^5$ and 3.7 times less likely to be classified into the Low-or-Intermediate Risk category, compared to a Low-or-Intermediate Risk drug, for the manual and hybrid dataset, respectively. These measures exceed or are very close to the “good” classification performance levels pre-defined by **Validation Strategy** (LR+ and 1/LR- > ~10 for excellent and >~5 for good performance).

Table 7. Likelihood Ratio Tests for the Two Thresholds for manual and hybrid validation datasets.

Validation dataset	Threshold	LR+	LR-
**Manual**	Threshold 1 (Low vs High/Intermediate)	4.5 (2.3 - 5)	0.11 (1.2e-06 - 0.23)
**Manual**	Threshold 2 (High vs Low/Intermediate)	12 (4.5 -1e+06)	1.1e-06 (1e-06 - 0.3)
**Hybrid**	Threshold 1 (Low vs High/Intermediate)	8.2e+5 (7.3e+5 - 1e+06)	0.18 (1e-06 - 0.27)
**Hybrid**	Threshold 2 (High vs Low/Intermediate)	6 (3 - 12)	0.27(1.1e-06 - 0.33)

Median and 95%CI of the LR+ and LR- values are shown. LR for positive results (LR+) indicates how much more likely a higher risk drug would be classified into the higher risk category compared to a lower risk drug, while the inverse of LR for negative results (LR-) indicates how much less likely a higher risk drug would be classified into the lower risk category compared to a lower risk drug.

Mean Classification Error as a Classification Performance Measure

For the calculation of mean classification error, the Low, Intermediate, and High Risk levels were given numerical categorical values 0, 1, and 2, respectively. Prediction was performed on each sample of the validation drugs, and the error was calculated as the absolute difference between the predicted and true risk category. Errors across all samples of all drugs were used to calculate the mean and standard deviation (SD) of the classification error. The 95% CI of the mean error was calculated as $mean \pm 1.96 * SD/\sqrt{N}$, where SD is the standard deviation of the error and N is the total number of samples (16 drugs x 2000 samples for validation). Comparing all *torsade metric score*s of the 16 validation drugs to the pre-defined thresholds, the mean classification error is 0.1974 (95%CI 0.1973-0.1975) and 0.2580 (0.2579-0.2581) for the manual and hybrid dataset respectively, both reaching the “excellent” performance level (< ~0.3) pre-defined by the Validation Strategy. A visualization of the relative position between *torsade*

metric score distributions and classification thresholds is provided in **Fig 4**. A quantification of the classification based on this relative position is provided in **Table 8**.

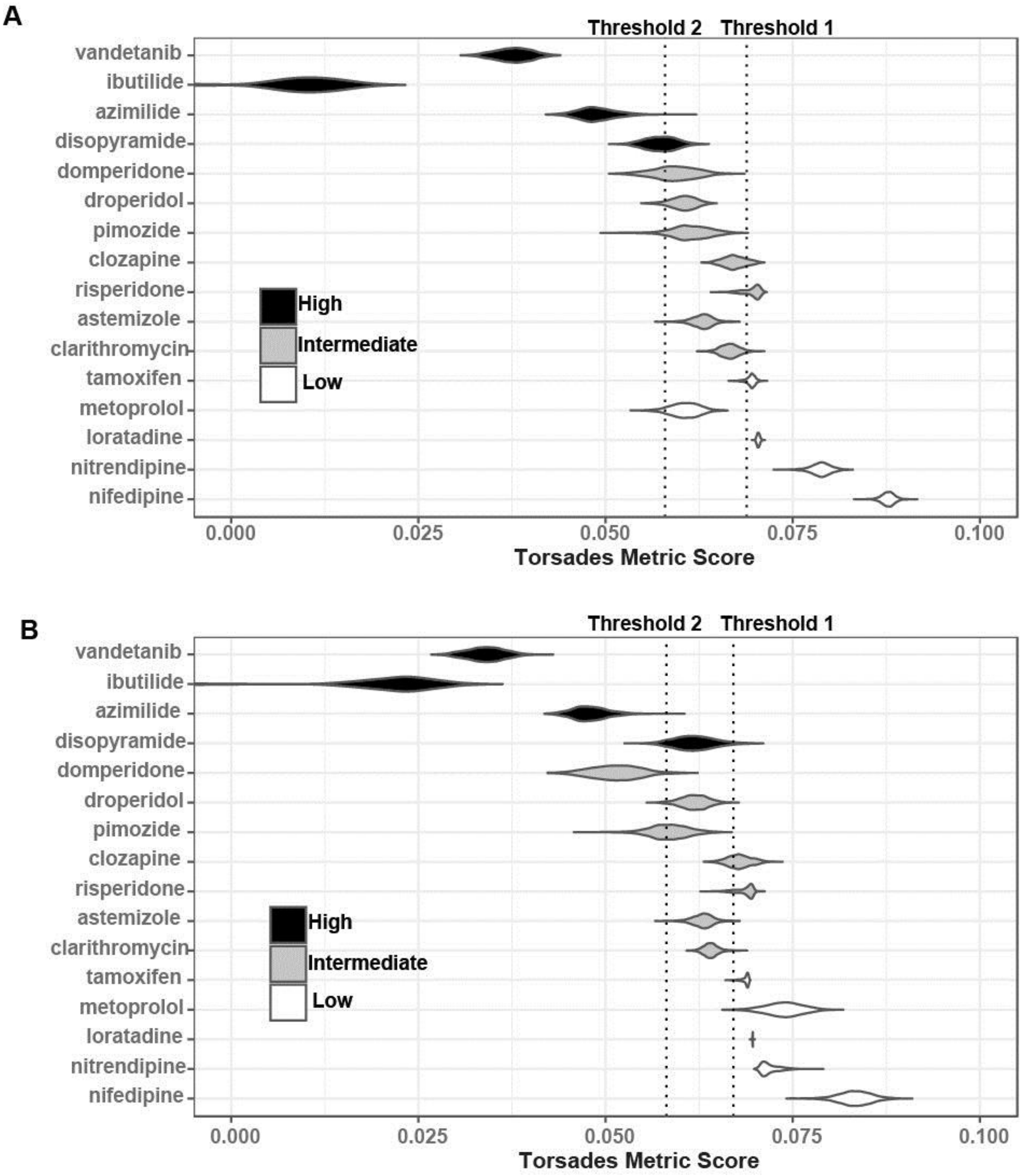

Figure 4. The distribution of *torsade metric score* values for the 16 CiPA validation drugs.

For each of the validation drugs, 2000 *torsade metric score*s are calculated using the UQ method developed earlier (1) to describe the probability distribution of the risk metric. The fraction of each drug's *torsade metric score* distribution predicted as High Risk (left to Threshold 2) is the probability this drug belongs to High Risk category, while the fraction predicted as Low (right to Threshold 1) or Intermediate (between Threshold 1 and 2) are the probabilities in Low or Intermediate Risk category respectively. Threshold 1 and Threshold 2 are pre-defined by training as in section 2 of this document. **A**) results for the manual validation dataset. **B**) the hybrid validation dataset.

Table 8. Distribution of *torsade metric score* across 3 TdP Risk Categories for Validation Drugs.

drug	class	Manual Dataset	Hybrid Dataset
**astemizole**	Intermediate	0.001,0.999,0	0.001,0.9945,0.0045
**azimilide**	High	0.9985,0.0015,0	0.9995,5e-04,0
**clarithromycin**	Intermediate	0,0.9695,0.0305	0,0.9935,0.0065
**clozapine**	Intermediate	0,0.8545,0.1455	0,0.3075,0.6925
**disopyramide**	High	0.5925,0.4075,0	0.08,0.886,0.034
**domperidone**	Intermediate	0.322,0.678,0	0.9775,0.0225,0
**droperidol**	Intermediate	0.0755,0.9245,0	0.029,0.9685,0.0025
**ibutilide**	High	1,0,0	1,0,0
**loratadine**	Low	0,0,1	0,0,1
**metoprolol**	Low	0.0805,0.9195,0	0,0.0035,0.9965
**nifedipine**	Low	0,0,1	0,0,1
**nitrendipine**	Low	0,0,1	0,0,1
**pimozide**	Intermediate	0.0785,0.921,5e-04	0.45,0.55,0
**risperidone**	Intermediate	0,0.2525,0.7475	0,0.137,0.863
**tamoxifen**	Low	0,0.1505,0.8495	0,0.0155,0.9845
**vandetanib**	High	1,0,0	1,0,0

For each drug, the distribution of its 2000 *torsade metric scores* across the 3 CiPA TdP risk categories is represented by 3 comma-separated numbers. The first number is the fraction classified as High Risk (left to Threshold 2), second one fraction of Intermediate Risk (between Threshold 1 and 2), and the last one fraction classified as Low Risk (right to Threshold 1). The distribution using the manual validation dataset is shown in column 3 while the hybrid dataset is shown in column 4.

Comparing To Alternative Metrics

The Leave-One-Out Cross Validation (LOOCV) to compare different metrics were done similarly to before (1). For each metric a probability distribution characterized by 2000 sample values per drug was estimated using the UQ method we developed for qNet/TMS above. An ordinal logistic regression classifier was trained on all samples from each metric using all but one left-out drug, and then was used to predict the TdP risk category of all 2000 samples of that left-out drug. As typically done for LOOCV, both ranking and classification were performed based on the predicted risk category probabilities. Of note this is different from the validation of the TMS metric as done in section 7, where ranking is based on the continuous TMS values directly, without introducing any statistical models to predict probabilities.

For ROC1 the ranking was based on the predicted probabilities in High or Intermediate Risk categories (P(High)+P(Intermediate) as the basis for ranking drugs), while ROC2 was based on the predicted probabilities in High category (P(High) as the basis for ranking drugs). For pairwise comparison the predicted ranking was compared to the true ranking of risk categories between two drugs in a pair. The predicted pairwise ranking was determined by following rules: if the two drugs are predicted into different categories (highest probabilities of two drugs are in two different categories), then ranking was based on predicted categories; if two drugs are both predicted into High category, then the predicted ranking was based on their probabilities in the High Risk category; if two drugs are both predicted into Intermediate or Low category, then the predicted ranking was based on their probabilities in the Low Risk category.

The Likelihood Ratio (LR) for LOOCV was calculated in the following manner. For Threshold 1, positive drugs are those whose highest predicted probability is in High or Intermediate category (P(High)> P(Low) or P(Intermediate)>P(Low)), while negative drugs are those predicted as Low Risk (P(Low) > P(High) and P(low) > P(Intermediate)). For Threshold 2, positive drugs are those whose highest predicted probability is in High Risk category (P(High)> P(Low) and P(High)>P(Intermediate)), while negative drugs are those predicted as Intermediate or Low Risk (P(Low) > P(High) or P(Intermediate) > P(High)). The mean classification error of LOOCV for 28 drugs was calculated the same way as the validation study using 16 drugs.

The comparison of qNet/*torsade metric score* to various alternative metrics are given in Tables 9-11 below. In Table 9 three metrics are compared: qNet/*torsade metric score*, APD90(16), and APD50&diastolic Ca concentration (17). Note that the latter two were originally developed using different experimental protocols and simulation/statistical models, whereas here all three are computed by CiPAORdv1.0 with drug binding dynamic parameters on hERG and block potency parameters on non-hERG currents. In Table 10 the same three metrics were compared, but they were computed by CiPAORdv1.0 model using drug block potency parameters (IC50s and Hill coefficients) for all 4 essential currents. For all the simulations in Tables 9&10, 1-4x Cmax was used for metric calculations. For Table 11 two simple statistical models were used without introducing any physiological models: the MICE model 5 used IC50s for ICaL and IKr/hERG (12), while the Bnet metric used IC50s and Hill coefficients to calculate the percentage of block of both depolarizing and repolarizing currents at 1x therapeutic free concentration (1x Cmax) (18). For calculation of the metrics in Tables 10-11, non-hERG block potency (IC50 and Hill coefficients) parameters for INaL, INa, and ICaL were listed in Tables 2&4 before. hERG block potency parameters were not listed before due to the use of dynamic hERG binding parameters (Table 3), so they are listed in Table 12 instead. The probabilities of being classified into each of

the three risk categories for all 28 drugs based on LOOCV are listed for various dataset and metric combinations in Tables 13-20.

Overall the CiPA metric (*torsamde metric score*) calculated by the CiPAORdv1.0 model (column 1 of Table 9) outperforms all other metrics (all other columns in Tables 9-11), especially on the measures that evaluate across all three categories (Pairwise Comparison Correct Rate for ranking, and Mean Classification Error for classification).

Table 9. Comparing to Other Metrics Calculated using hERG Dynamic Data and non-hERG Potency Data.

Performance Measure	Dataset	qNet/*torsade metric score*	APD90	APD50&Diastolic Ca
AUC of ROC1	Manual	0.901 (0.883 - 0.924)	0.842 (0.801 - 0.877)	0.854 (0.825 - 0.889)
	Hybrid	0.971 (0.936 - 1)	0.848 (0.807 - 0.889)	0.854 (0.807 - 0.906)
AUC of ROC2	Manual	0.988 (0.95 - 1)	0.975 (0.962 - 0.988)	0.988 (0.944 - 1)
	Hybrid	0.919 (0.869 - 0.962)	0.975 (0.956 - 0.981)	0.969 (0.925 - 0.981)
Pairwise Comparison Correct Rate	Manual	0.929 (0.905 - 0.943)	0.886 (0.858 - 0.91)	0.891 (0.829 - 0.924)
	Hybrid	0.943 (0.905 - 0.976)	0.891 (0.863 - 0.919)	0.896 (0.858 - 0.929)
LR+ of Threshold 1	Manual	8.05 (4.03 - 9)	2.53 (1.89 - 2.84)	4.03 (2.68 - 4.26)
	Hybrid	8.05 (4.03 - 9.47e+05)	2.68 (2.01 - 4.03)	3.55 (2.37 - 4.26)
LR- of Threshold 1	Manual	0.0677 (1.13e-06 - 0.178)	0.189 (0.0789 - 0.316)	0.135 (0.0677 - 0.203)
	Hybrid	0.0677 (1.12e-06 - 0.158)	0.158 (0.0789 - 0.284)	0.203 (0.0789 - 0.316)
LR+ of Threshold 2	Manual	7.5e+05 (8.75 - 1e+06)	15 (12.5 - 17.5)	17.5 (15 - 8.75e+05)
	Hybrid	15 (6.25 - 17.5)	15 (12.5 - 17.5)	15 (7.5 - 17.5)

**LR- of Threshold 2**	Manual	0.25 (1e-06 - 0.263)	0.263 (0.132 - 0.395)	0.25 (1.05e-06 - 0.263)
	Hybrid	0.263 (0.132 - 0.395)	0.263 (0.132 - 0.395)	0.263 (0.132 - 0.395)
**Mean Classification Error**	Manual	0.158 (0.155 - 0.161)	0.305 (0.301 - 0.309)	0.224 (0.221 - 0.228)
	Hybrid	0.203 (0.201 - 0.208)	0.285 (0.281 - 0.289)	0.291 (0.287 - 0.295)

For each performance measure (row), the values for three metrics (qNet/*torsade metric score*, APD90, APD50&diastolic Ca^{2+}) using the two datasets (manual and hybrid) are shown. All 28 CiPA drugs are used, with leave-one-out cross validation to calculate the performance measure. The median as well as 95% CI values for each performance measure are listed.

Table 10. Comparing to Other Metrics Calculated Without hERG Dynamic Data

Performance Measure	Dataset	qNet/*torsade metric score*	APD90	APD50&Diastolic Ca
**AUC of ROC1**	Manual (Crumb et al.)	0.86 (0.819 - 0.895)	0.784 (0.708 - 0.825)	0.76 (0.696 - 0.801)
	HTS (Site 6)	0.626 (0.579 - 0.684)	0.485 (0.439 - 0.538)	0.591 (0.509 - 0.702)
**AUC of ROC2**	Manual (Crumb et al.)	0.856 (0.812 - 0.9)	0.838 (0.781 - 0.881)	0.831 (0.787 - 0.881)
	HTS (Site 6)	0.756 (0.65 - 0.844)	0.666 (0.575 - 0.744)	0.791 (0.672 - 0.866)
**Pairwise Comparison Correct Rate**	Manual (Crumb et al.)	0.848 (0.81 - 0.877)	0.81 (0.763 - 0.844)	0.791 (0.744 - 0.825)
	HTS (Site 6)	0.73 (0.682 - 0.773)	0.635 (0.597 - 0.673)	0.706 (0.63 - 0.768)
**LR+ of Threshold 1**	Manual (Crumb et al.)	2.01 (1.61 - 2.84)	2.37 (1.66 - 2.68)	1.52 (1.26 - 2.01)
	HTS (Site 6)	1.08 (1.02 - 1.34)	0.711 (0.651 - 0.812)	1.22 (0.947 - 1.61)
**LR- of Threshold 1**	Manual (Crumb et al.)	0.118 (1.8e-06 - 0.284)	0.316 (0.158 - 0.474)	0.355 (0.118 - 0.592)
	HTS (Site 6)	0.711 (0.237 - 0.947)	3.32 (1.66 - 3.79)	0.474 (4.5e-06 - 1.42)
**LR+ of Threshold 2**	Manual (Crumb et al.)	5 (3.33 - 12.5)	6.25 (3.33 - 12.5)	5 (3.33 - 12.5)
	HTS (Site 6)	6.25 (2.5 - 12.5)	3.75 (1.67 - 10)	5 (2.5 - 10)
	Manual (Crumb et al.)	0.556 (0.278 - 0.588)	0.526 (0.395 - 0.588)	0.556 (0.395 - 0.588)

**LR- of Threshold 2**	HTS (Site 6)	0.556 (0.395 - 0.833)	0.694 (0.526 - 0.882)	0.441 (0.417 - 0.735)
**Mean Classification Error**	Manual (Crumb et al.)	0.403 (0.398 - 0.407)	0.435 (0.43 - 0.439)	0.479 (0.474 - 0.483)
	HTS (Site 6)	0.549 (0.545 - 0.553)	0.779 (0.775 - 0.784)	0.516 (0.511 - 0.521)

This table is calculated exactly the same way as Table 9 except that for hERG block, drug potency parameters (IC50s and Hill coefficients) were used in place of binding dynamic parameters. The hERG block potency parameters can be found in Table 12 and non-hERG block potency parameters were listed in Tables 2&4 before. For the manual dataset, the hERG potency parameters were calculated from Crumb et al.(5). For the HTS dataset, the hERG potency parameters were calculated from Site 6 high throughput data. Note that other than the hERG data used being different, the manual and HTS dataset in this table are the same as the manual and hybrid dataset in Table 9.

Table 11. Comparing to Other Metrics using Simple Statistical but not Physiological Models

**Performance Measure**	**Dataset**	**MICE**	**Bnet**
**AUC of ROC1**	Manual (Crumb et al.)	0.743 (0.713 - 0.795)	0.877 (0.83 - 0.924)
	HTS (Site 6)	0.708 (0.614 - 0.801)	0.637 (0.556 - 0.725)
**AUC of ROC2**	Manual (Crumb et al.)	0.7 (0.644 - 0.75)	0.856 (0.787 - 0.906)
	HTS (Site 6)	0.619 (0.475 - 0.738)	0.744 (0.631 - 0.85)
**Pairwise Comparison Correct Rate**	Manual (Crumb et al.)	0.73 (0.701 - 0.754)	0.853 (0.81 - 0.896)
	HTS (Site 6)	0.682 (0.607 - 0.744)	0.735 (0.668 - 0.796)
**LR+ of Threshold 1**	Manual (Crumb et al.)	1.61 (1.61 - 2.01)	2.68 (1.71 - 2.84)
	HTS (Site 6)	1.18 (0.947 - 1.66)	1.29 (1.08 - 1.71)
**LR- of Threshold 1**	Manual (Crumb et al.)	0.237 (0.189 - 0.237)	0.158 (0.0789 - 0.237)
	HTS (Site 6)	0.632 (0.355 - 1.11)	0.237 (3e-06 - 0.711)
	Manual (Crumb et al.)	1.67	10

**LR+ of Threshold 2**		(0.833 - 5)	(5 - 15)
	HTS (Site 6)	1.67<br>(5e-06 - 7.5)	6.25<br>(3.75 - 12.5)
**LR- of Threshold 2**	Manual (Crumb et al.)	0.882<br>(0.694 - 1.03)	0.526<br>(0.375 - 0.556)
	HTS (Site 6)	0.882<br>(0.588 - 1.25)	0.526<br>(0.395 - 0.694)
**Mean Classification Error**	Manual (Crumb et al.)	0.554<br>(0.549 - 0.559)	0.357<br>(0.353 - 0.361)
	HTS (Site 6)	0.697<br>(0.693 - 0.702)	0.472<br>(0.467 - 0.476)

This table is calculated similarly to Tables 9&10, with metrics computed by ordinal logistic models without the use of any physiological model. For MICE model 5, the input of the logistic regression model is log(IC50 of hERG) – log(IC50 of ICaL); for Bnet, the input is %block on hERG – (%block on INaL + %block on INa + %block on ICaL), calculated on each drug's 1x Cmax.

Table 12. Block potency parameters for hERG.

Drug	IC50 (Manual Crumb et al.)	Hill (Manual Crumb et al.)	IC50 (HTS Site 6)	Hill (HTS Site 6)
dofetilide	0.00147 (0.00119, 0.00179)	0.63 (0.49, 0.79)	0.0134 (0.00962, 0.0201)	1.4 (0.95, 7.5)
bepridil	0.15 (0.0893, 0.239)	0.93 (0.6, 1.9)	0.264 (0.213, 0.363)	1.4 (0.93, 2.2)
sotalol	88 (55.8, 129)	0.97 (0.71, 1.5)	281 (214 , 425)	1.2 (0.76, 1.8)
quinidine	0.34 (0.229, 0.441)	1 (0.69, 1.5)	0.792 (0.603, 1.1)	0.99 (0.75, 1.3)
cisapride	0.0118 (0.0104, 0.0136)	1.3 (1.1, 1.6)	0.071 (0.0534, 0.0872)	1.9 (1.2, 8.6)
terfenadine	0.0183 (0.011, 0.0304)	0.6 (0.44, 0.86)	0.163 (0.0914, 0.417)	0.93 (0.44, 1.8)
ondansetron	1.5 (1 , 2.22)	0.99 (0.7, 1.5)	0.873 (0.727, 1.04)	1 (0.87, 1.3)
chlorpromazine	1.12 (0.824, 1.53)	0.9 (0.7, 1.2)	0.85 (0.647, 1.15)	1.7 (1.1, 4.7)
verapamil	0.502 (0.43, 0.59)	1.1 (0.89, 1.3)	0.172 (0.144, 0.203)	1.3 (1.1, 1.6)
ranolazine	6.52 (4.97, 8.85)	0.84 (0.65, 1.1)	3.38 (2.53, 4.18)	1.1 (0.84, 1.5)
mexiletine	21.2 (12.8, 66.7)	3.1 (1.3, 9.3)	36.7 (30 , 45.3)	1 (0.82, 1.3)
diltiazem	6.66 (5.17, 8.49)	0.8 (0.67, 0.99)	4.19 (3 , 5.45)	0.86 (0.66, 1.1)
vandetanib	0.251 (0.203, 0.318)	0.81 (0.7, 0.96)	0.377 (0.167, 1.47)	1.1 (0.49, 8.4)
ibutilide	0.0146 (0.0121, 0.0175)	1.1 (0.89, 1.5)	0.0011 (5.62e-13, 0.00712)	4.2 (0.53, 9.8)
azimilide	0.828 (0.614, 1.12)	0.75 (0.61, 0.95)	0.12 (0.103, 0.141)	1 (0.91, 1.2)

disopyramide	11.2 (9.11, 13.8)	0.85 (0.72, 1)	0.902 (0.693, 1.19)	0.95 (0.76, 1.2)
domperidone	0.132 (0.105, 0.166)	0.78 (0.66, 0.93)	0.0201 (0.0182, 0.0224)	1.6 (1.1, 8.6)
droperidol	0.0403 (0.0328, 0.0499)	1.1 (0.93, 1.4)	0.0428 (0.0373, 0.0487)	1.1 (1 , 1.2)
pimozide	0.0344 (0.0242, 0.0474)	0.71 (0.57, 0.9)	0.0449 (0.0423, 0.0523)	3.5 (1.4, 9.6)
clozapine	2.63 (2.16, 3.16)	0.73 (0.64, 0.84)	0.837 (0.75, 1.14)	3.9 (1.6, 9.6)
risperidone	0.139 (0.0849, 0.232)	0.68 (0.51, 0.98)	0.0788 (0.0624, 0.0995)	1.1 (0.94, 1.4)
astemizole	0.00999 (0.00596, 0.0171)	0.54 (0.44, 0.68)	0.0233 (0.00445, 0.189)	5.4 (1.5, 9.8)
clarithromycin	62.1 (48.9, 78.8)	0.72 (0.61, 0.86)	21.3 (16.6, 27.4)	0.81 (0.68, 0.95)
tamoxifen	3.49 (2.63, 4.62)	0.78 (0.63, 0.99)	2.12 (1.96, 2.3)	4.9 (2 , 9.6)
metoprolol	20.1 (16.7, 24.2)	0.89 (0.76, 1.1)	13.6 (11.1, 16.7)	0.96 (0.82, 1.1)
loratadine	27.2 (21.6, 34.2)	0.63 (0.53, 0.82)	0.676 (0.518, 1.62)	3.5 (1.2, 9.6)
nitrendipine	19.9 (12.4, 46.1)	0.53 (0.3, 0.89)	11.2 (7.66, 24)	1.5 (1.1, 6.3)
nifedipine	142 (122 , 165)	0.75 (0.69, 0.8)	29.1 (22.5, 37.9)	1.1 (0.8, 1.6)

The median values and 95% Credible Intervals (CIs) for IC50s and Hill coefficients obtained using Markov-chain Monte Carlo simulation for both training and validation drugs. For clarity the values are rounded to limited numbers of significant digits. The unit for IC50s here is µM. During simulation the IC50s were converted to nM to match previous simulations.

Table 13. CiPA drugs' probabilities of being classified into the 3 TdP Risk categories based on LOOCV of qNet/*torsade metric score* with hERG dynamic data.

Drug	True Risk	Manual Dataset			Hybrid Dataset		
		p(Low)	p(Inter)	p(High)	p(Low)	p(Inter)	p(High)
**quinidine**	High	0.0000	0.0000	1.0000	0.0000	0.1300	0.8700
**bepridil**	High	0.0000	0.0000	1.0000	0.0000	0.0000	1.0000
**dofetilide**	High	0.0000	0.0035	0.9965	0.0000	0.0005	0.9995
**sotalol**	High	0.0000	0.6480	0.3520	0.0000	0.6645	0.3355
**ibutilide**	High	0.0000	0.0000	1.0000	0.0000	0.0000	1.0000
**azimilide**	High	0.0000	0.0035	0.9965	0.0000	0.0015	0.9985
**disopyramide**	High	0.0000	0.8325	0.1675	0.0145	0.9840	0.0015
**vandetanib**	High	0.0000	0.0000	1.0000	0.0000	0.0000	1.0000
**chlorpromazine**	Intermediate	0.0145	0.9855	0.0000	0.0285	0.9715	0.0000
**cisapride**	Intermediate	0.0000	1.0000	0.0000	0.0000	1.0000	0.0000
**terfenadine**	Intermediate	0.0000	0.9960	0.0040	0.0000	0.9995	0.0005
**ondansetron**	Intermediate	0.0000	1.0000	0.0000	0.0075	0.9925	0.0000
**risperidone**	Intermediate	0.9135	0.0865	0.0000	0.7630	0.2370	0.0000
**domperidone**	Intermediate	0.0005	0.8025	0.1970	0.0000	0.0220	0.9780
**clarithromycin**	Intermediate	0.1150	0.8850	0.0000	0.0005	0.9995	0.0000
**astemizole**	Intermediate	0.0000	0.9655	0.0345	0.0000	1.0000	0.0000
**droperidol**	Intermediate	0.0000	0.9815	0.0185	0.0000	0.9980	0.0020
**pimozide**	Intermediate	0.0005	0.9680	0.0315	0.0000	0.7630	0.2370
**clozapine**	Intermediate	0.2955	0.7045	0.0000	0.4730	0.5270	0.0000
**diltiazem**	Low	1.0000	0.0000	0.0000	1.0000	0.0000	0.0000
**mexiletine**	Low	1.0000	0.0000	0.0000	1.0000	0.0000	0.0000
**ranolazine**	Low	0.9995	0.0005	0.0000	1.0000	0.0000	0.0000
**verapamil**	Low	0.9980	0.0020	0.0000	0.3110	0.6890	0.0000
**metoprolol**	Low	0.0000	0.9535	0.0465	0.9820	0.0180	0.0000
**tamoxifen**	Low	0.7495	0.2505	0.0000	0.2885	0.7115	0.0000
**loratadine**	Low	1.0000	0.0000	0.0000	1.0000	0.0000	0.0000
**nifedipine**	Low	1.0000	0.0000	0.0000	1.0000	0.0000	0.0000
**nitrendipine**	Low	1.0000	0.0000	0.0000	1.0000	0.0000	0.0000

For each of the 28 drugs, the fractions of its 2000 samples classified into the three CiPA TdP risk categories using qNet/*torsade metric scores* based on LOOCV is given by p(Low), p(Inter), and p(High) respectively. Simulation procedure is the same as Table 9 (used hERG dynamic data).

Table 14. CiPA drugs' probabilities of being classified into the 3 TdP Risk categories based on LOOCV of the APD90 metric using hERG dynamic data.

Drug	True Risk	Manual Dataset			Hybrid Dataset		
		p(Low)	p(Inter)	p(High)	p(Low)	p(Inter)	p(High)
**quinidine**	High	0.0000	0.0000	1.0000	0.0000	0.0000	1.0000
**bepridil**	High	0.0000	0.0000	1.0000	0.0000	0.0000	1.0000
**dofetilide**	High	0.0000	0.3265	0.6735	0.0000	0.2165	0.7835
**sotalol**	High	0.0000	0.9860	0.0140	0.0000	0.9795	0.0205
**ibutilide**	High	0.0000	0.0000	1.0000	0.0000	0.0000	1.0000
**azimilide**	High	0.0000	0.0095	0.9905	0.0000	0.0060	0.9940
**disopyramide**	High	0.0000	0.5630	0.4370	0.0000	0.7625	0.2375
**vandetanib**	High	0.0000	0.0000	1.0000	0.0000	0.0000	1.0000
**chlorpromazine**	Intermediate	0.2750	0.7250	0.0000	0.3515	0.6485	0.0000
**cisapride**	Intermediate	0.0000	1.0000	0.0000	0.0000	1.0000	0.0000
**terfenadine**	Intermediate	0.0000	1.0000	0.0000	0.0000	1.0000	0.0000
**ondansetron**	Intermediate	0.0000	1.0000	0.0000	0.0000	1.0000	0.0000
**risperidone**	Intermediate	0.9995	0.0005	0.0000	0.9995	0.0005	0.0000
**domperidone**	Intermediate	0.0000	0.0235	0.9765	0.0000	0.0035	0.9965
**clarithromycin**	Intermediate	0.0960	0.9040	0.0000	0.0200	0.9800	0.0000
**astemizole**	Intermediate	0.0155	0.9845	0.0000	0.0180	0.9820	0.0000
**droperidol**	Intermediate	0.0000	1.0000	0.0000	0.0005	0.9995	0.0000
**pimozide**	Intermediate	0.0020	0.9880	0.0100	0.0000	0.9840	0.0160
**clozapine**	Intermediate	0.8950	0.1050	0.0000	0.9280	0.0720	0.0000
**diltiazem**	Low	1.0000	0.0000	0.0000	1.0000	0.0000	0.0000
**mexiletine**	Low	0.6070	0.3930	0.0000	0.9020	0.0980	0.0000
**ranolazine**	Low	0.0000	1.0000	0.0000	0.0000	1.0000	0.0000
**verapamil**	Low	0.0075	0.9915	0.0010	0.0000	0.9985	0.0015
**metoprolol**	Low	0.0020	0.9980	0.0000	0.4160	0.5840	0.0000
**tamoxifen**	Low	1.0000	0.0000	0.0000	1.0000	0.0000	0.0000
**loratadine**	Low	1.0000	0.0000	0.0000	1.0000	0.0000	0.0000
**nifedipine**	Low	1.0000	0.0000	0.0000	1.0000	0.0000	0.0000
**nitrendipine**	Low	1.0000	0.0000	0.0000	1.0000	0.0000	0.0000

For each of the 28 drugs, the fractions of its 2000 samples classified into the three CiPA TdP risk categories using APD90 are given by p(Low), p(Inter), and p(High) respectively. Simulation procedure is the same as Table 9 (used hERG dynamic data).

Table 15. CiPA drugs' probabilities of being classified into the 3 TdP Risk categories based on LOOCV using the metric of APD50 plus diastolic Ca with hERG dynamic data.

Drug	True Risk	Manual Dataset			Hybrid Dataset		
		p(Low)	p(Inter)	p(High)	p(Low)	p(Inter)	p(High)
**quinidine**	High	0.0000	0.0000	1.0000	0.0000	0.0000	1.0000
**bepridil**	High	0.0000	0.0000	1.0000	0.0000	0.0000	1.0000
**dofetilide**	High	0.0000	0.0000	1.0000	0.0000	0.0365	0.9635
**sotalol**	High	0.0000	0.8350	0.1650	0.0000	0.9920	0.0080
**ibutilide**	High	0.0000	0.0000	1.0000	0.0000	0.0000	1.0000
**azimilide**	High	0.0000	0.0035	0.9965	0.0000	0.0055	0.9945
**disopyramide**	High	0.0000	0.7855	0.2145	0.0000	0.9070	0.0930
**vandetanib**	High	0.0000	0.0000	1.0000	0.0000	0.0000	1.0000
**chlorpromazine**	Intermediate	0.0120	0.9880	0.0000	0.1745	0.8255	0.0000
**cisapride**	Intermediate	0.0000	1.0000	0.0000	0.0000	1.0000	0.0000
**terfenadine**	Intermediate	0.0000	1.0000	0.0000	0.0000	1.0000	0.0000
**ondansetron**	Intermediate	0.0000	1.0000	0.0000	0.7015	0.2985	0.0000
**risperidone**	Intermediate	0.9910	0.0090	0.0000	0.9940	0.0060	0.0000
**domperidone**	Intermediate	0.0000	0.4105	0.5895	0.0000	0.0010	0.9990
**clarithromycin**	Intermediate	0.2895	0.7105	0.0000	0.0095	0.9905	0.0000
**astemizole**	Intermediate	0.0000	0.9955	0.0045	0.0055	0.9945	0.0000
**droperidol**	Intermediate	0.0000	0.9960	0.0040	0.0000	1.0000	0.0000
**pimozide**	Intermediate	0.0130	0.9720	0.0150	0.0000	0.9670	0.0330
**clozapine**	Intermediate	0.6825	0.3175	0.0000	0.8430	0.1570	0.0000
**diltiazem**	Low	1.0000	0.0000	0.0000	1.0000	0.0000	0.0000
**mexiletine**	Low	0.9825	0.0175	0.0000	0.9900	0.0100	0.0000
**ranolazine**	Low	0.0000	0.9965	0.0035	0.0055	0.9945	0.0000
**verapamil**	Low	0.9935	0.0065	0.0000	0.0155	0.9835	0.0010
**metoprolol**	Low	0.0000	0.9925	0.0075	0.5400	0.4600	0.0000
**tamoxifen**	Low	0.9810	0.0190	0.0000	0.9985	0.0015	0.0000
**loratadine**	Low	1.0000	0.0000	0.0000	1.0000	0.0000	0.0000
**nifedipine**	Low	1.0000	0.0000	0.0000	1.0000	0.0000	0.0000
**nitrendipine**	Low	1.0000	0.0000	0.0000	1.0000	0.0000	0.0000

For each of the 28 drugs, the fractions of its 2000 samples classified into the three CiPA TdP risk categories using APD50 plus diastolic Ca are given by p(Low), p(Inter), and p(High) respectively. Simulation procedure is the same as Table 9 (used hERG dynamic data).

Table 16. CiPA drugs' probabilities of being classified into the 3 TdP Risk categories based on LOOCV using the metric of qNet/*torsade metric score* without hERG dynamic data.

Drug	True Risk	Manual (Crumb et al.)			HTS (Site 6)		
		p(Low)	p(Inter)	p(High)	p(Low)	p(Inter)	p(High)
**quinidine**	High	0.0000	0.0000	1.0000	0.0000	0.8160	0.1840
**bepridil**	High	0.0025	0.7430	0.2545	0.0135	0.9865	0.0000
**dofetilide**	High	0.0000	0.0000	1.0000	0.0000	1.0000	0.0000
**sotalol**	High	0.0000	0.8265	0.1735	0.0000	1.0000	0.0000
**ibutilide**	High	0.0000	0.0000	1.0000	0.0000	0.0000	1.0000
**azimilide**	High	0.0000	1.0000	0.0000	0.0000	0.0015	0.9985
**disopyramide**	High	0.0005	0.9995	0.0000	0.0000	0.2840	0.7160
**vandetanib**	High	0.0000	0.0000	1.0000	0.0015	0.2120	0.7865
**chlorpromazine**	Intermediate	0.0700	0.9300	0.0000	0.6530	0.3470	0.0000
**cisapride**	Intermediate	0.0000	0.1270	0.8730	0.0075	0.9925	0.0000
**terfenadine**	Intermediate	0.0000	0.0180	0.9820	0.5155	0.4845	0.0000
**ondansetron**	Intermediate	0.0005	0.9970	0.0025	0.0000	1.0000	0.0000
**risperidone**	Intermediate	0.0335	0.9665	0.0000	0.0015	0.9985	0.0000
**domperidone**	Intermediate	0.3525	0.6475	0.0000	0.0000	0.0000	1.0000
**clarithromycin**	Intermediate	0.1830	0.8170	0.0000	0.0000	1.0000	0.0000
**astemizole**	Intermediate	0.0000	1.0000	0.0000	0.0000	1.0000	0.0000
**droperidol**	Intermediate	0.0000	0.9080	0.0920	0.0000	1.0000	0.0000
**pimozide**	Intermediate	0.7865	0.2135	0.0000	0.6075	0.3925	0.0000
**clozapine**	Intermediate	0.0000	1.0000	0.0000	0.8155	0.1845	0.0000
**diltiazem**	Low	1.0000	0.0000	0.0000	0.0000	1.0000	0.0000
**mexiletine**	Low	0.9990	0.0010	0.0000	1.0000	0.0000	0.0000
**ranolazine**	Low	0.0815	0.9185	0.0000	0.0000	1.0000	0.0000
**verapamil**	Low	0.8060	0.1940	0.0000	0.0000	0.4725	0.5275
**metoprolol**	Low	0.0000	0.7855	0.2145	0.0095	0.9905	0.0000
**tamoxifen**	Low	0.0000	1.0000	0.0000	0.0000	1.0000	0.0000
**loratadine**	Low	0.0040	0.9960	0.0000	0.0000	1.0000	0.0000
**nifedipine**	Low	1.0000	0.0000	0.0000	1.0000	0.0000	0.0000
**nitrendipine**	Low	1.0000	0.0000	0.0000	0.0760	0.9240	0.0000

For each of the 28 drugs, the fractions of its 2000 samples classified into the three CiPA TdP risk categories using qNet/*torsade metric score* are given by p(Low), p(Inter), and p(High) respectively. Simulation procedure is the same as Table 10.

Table 17. CiPA drugs' probabilities of being classified into the 3 TdP Risk categories based on LOOCV using the metric of APD90 without hERG dynamic data.

Drug	True Risk	Manual (Crumb et al.)			HTS (Site 6)		
		p(Low)	p(Inter)	p(High)	p(Low)	p(Inter)	p(High)
**quinidine**	High	0.0000	0.0000	1.0000	0.0000	0.0000	1.0000
**bepridil**	High	0.0040	0.8010	0.1950	0.0055	0.9945	0.0000
**dofetilide**	High	0.0000	0.0000	1.0000	0.0400	0.9600	0.0000
**sotalol**	High	0.0025	0.9600	0.0375	0.0445	0.9555	0.0000
**ibutilide**	High	0.0000	0.0000	1.0000	0.0000	0.0000	1.0000
**azimilide**	High	0.0000	1.0000	0.0000	0.0000	0.9810	0.0190
**disopyramide**	High	0.0055	0.9945	0.0000	0.0000	0.4900	0.5100
**vandetanib**	High	0.0000	0.0000	1.0000	0.0125	0.4440	0.5435
**chlorpromazine**	Intermediate	0.7250	0.2750	0.0000	0.9955	0.0045	0.0000
**cisapride**	Intermediate	0.0000	0.9405	0.0595	0.9965	0.0035	0.0000
**terfenadine**	Intermediate	0.0000	0.0440	0.9560	0.7795	0.2205	0.0000
**ondansetron**	Intermediate	0.0410	0.9585	0.0005	0.0000	1.0000	0.0000
**risperidone**	Intermediate	0.7365	0.2635	0.0000	0.9990	0.0010	0.0000
**domperidone**	Intermediate	0.0005	0.9995	0.0000	0.0000	0.0000	1.0000
**clarithromycin**	Intermediate	0.8865	0.1135	0.0000	0.0000	1.0000	0.0000
**astemizole**	Intermediate	0.0005	0.9995	0.0000	1.0000	0.0000	0.0000
**droperidol**	Intermediate	0.0000	0.9985	0.0015	0.0000	1.0000	0.0000
**pimozide**	Intermediate	0.9855	0.0145	0.0000	1.0000	0.0000	0.0000
**clozapine**	Intermediate	0.1835	0.8165	0.0000	0.9990	0.0010	0.0000
**diltiazem**	Low	1.0000	0.0000	0.0000	0.0000	1.0000	0.0000
**mexiletine**	Low	0.7890	0.1950	0.0160	0.0665	0.9335	0.0000
**ranolazine**	Low	0.0000	0.4350	0.5650	0.0000	0.5755	0.4245
**verapamil**	Low	0.0005	0.9995	0.0000	0.0000	0.3490	0.6510
**metoprolol**	Low	0.0000	1.0000	0.0000	0.0000	1.0000	0.0000
**tamoxifen**	Low	0.9800	0.0200	0.0000	0.0000	1.0000	0.0000
**loratadine**	Low	1.0000	0.0000	0.0000	0.0000	1.0000	0.0000
**nifedipine**	Low	1.0000	0.0000	0.0000	0.9845	0.0155	0.0000
**nitrendipine**	Low	1.0000	0.0000	0.0000	0.0005	0.9995	0.0000

For each of the 28 drugs, the fractions of its 2000 samples classified into the three CiPA TdP risk categories using APD90 are given by p(Low), p(Inter), and p(High) respectively. Simulation procedure is the same as Table 10.

Table 18. CiPA drugs' probabilities of being classified into the 3 TdP Risk categories based on LOOCV using the metric of APD50 plus diastolic Ca without hERG dynamic data.

Drug	True Risk	Manual (Crumb et al.)			HTS (Site 6)		
		p(Low)	p(Inter)	p(High)	p(Low)	p(Inter)	p(High)
**quinidine**	High	0.0000	0.0000	1.0000	0.0140	0.1675	0.8185
**bepridil**	High	0.0025	0.8580	0.1395	0.0425	0.9575	0.0000
**dofetilide**	High	0.0000	0.0000	1.0000	0.0000	1.0000	0.0000
**sotalol**	High	0.0000	0.9450	0.0550	0.0015	0.9985	0.0000
**ibutilide**	High	0.0000	0.0000	1.0000	0.0000	0.0000	1.0000
**azimilide**	High	0.0000	1.0000	0.0000	0.0000	0.0000	1.0000
**disopyramide**	High	0.0040	0.9960	0.0000	0.0235	0.1920	0.7845
**vandetanib**	High	0.0000	0.0000	1.0000	0.0015	0.0980	0.9005
**chlorpromazine**	Intermediate	0.0885	0.9115	0.0000	0.3030	0.6970	0.0000
**cisapride**	Intermediate	0.0000	0.6845	0.3155	0.0020	0.9980	0.0000
**terfenadine**	Intermediate	0.0000	0.0460	0.9540	0.0610	0.9385	0.0005
**ondansetron**	Intermediate	0.0010	0.9975	0.0015	0.8750	0.1245	0.0005
**risperidone**	Intermediate	0.1755	0.8245	0.0000	0.0000	1.0000	0.0000
**domperidone**	Intermediate	0.9320	0.0680	0.0000	0.0000	0.0000	1.0000
**clarithromycin**	Intermediate	0.4805	0.5195	0.0000	0.0090	0.9910	0.0000
**astemizole**	Intermediate	0.0000	1.0000	0.0000	0.0000	1.0000	0.0000
**droperidol**	Intermediate	0.0000	0.9900	0.0100	0.0000	1.0000	0.0000
**pimozide**	Intermediate	0.9420	0.0580	0.0000	0.2980	0.7020	0.0000
**clozapine**	Intermediate	0.0010	0.9990	0.0000	0.2770	0.7230	0.0000
**diltiazem**	Low	1.0000	0.0000	0.0000	0.0095	0.9905	0.0000
**mexiletine**	Low	0.8175	0.1810	0.0015	0.7575	0.2425	0.0000
**ranolazine**	Low	0.0000	0.4260	0.5740	0.0060	0.0600	0.9340
**verapamil**	Low	0.0645	0.9355	0.0000	0.1005	0.4440	0.4555
**metoprolol**	Low	0.0000	0.9980	0.0020	0.0285	0.9715	0.0000
**tamoxifen**	Low	0.0000	1.0000	0.0000	0.0000	1.0000	0.0000
**loratadine**	Low	0.0030	0.9970	0.0000	0.0000	1.0000	0.0000
**nifedipine**	Low	1.0000	0.0000	0.0000	1.0000	0.0000	0.0000
**nitrendipine**	Low	1.0000	0.0000	0.0000	0.4410	0.5590	0.0000

For each of the 28 drugs, the fractions of its 2000 samples classified into the three CiPA TdP risk categories using APD50 plus diastolic Ca are given by p(Low), p(Inter), and p(High) respectively. Simulation procedure is the same as Table 10.

Table 19. CiPA drugs' probabilities of being classified into the 3 TdP Risk categories based on LOOCV using the MICE model.

Drug	True Risk	Manual (Crumb et al.)			HTS (Site 6)		
		p(Low)	p(Inter)	p(High)	p(Low)	p(Inter)	p(High)
**quinidine**	High	0.0000	0.9180	0.0820	0.0000	0.6050	0.3950
**bepridil**	High	0.0000	1.0000	0.0000	0.1645	0.4695	0.3660
**dofetilide**	High	0.0000	0.2740	0.7260	0.0000	0.3865	0.6135
**sotalol**	High	0.0000	0.9955	0.0045	0.1755	0.8050	0.0195
**ibutilide**	High	0.0000	0.0000	1.0000	0.0000	0.4260	0.5740
**azimilide**	High	0.0000	1.0000	0.0000	0.0000	1.0000	0.0000
**disopyramide**	High	0.0000	1.0000	0.0000	0.0000	0.9765	0.0235
**vandetanib**	High	0.0000	1.0000	0.0000	0.4715	0.5285	0.0000
**chlorpromazine**	Intermediate	0.0000	1.0000	0.0000	0.6845	0.2340	0.0815
**cisapride**	Intermediate	0.0000	0.0570	0.9430	0.0180	0.3130	0.6690
**terfenadine**	Intermediate	0.0000	0.9985	0.0015	0.1375	0.3370	0.5255
**ondansetron**	Intermediate	0.0000	0.9995	0.0005	0.0000	0.4185	0.5815
**risperidone**	Intermediate	0.0000	1.0000	0.0000	0.0000	0.3090	0.6910
**domperidone**	Intermediate	0.9955	0.0045	0.0000	0.0000	1.0000	0.0000
**clarithromycin**	Intermediate	0.9970	0.0030	0.0000	0.9895	0.0105	0.0000
**astemizole**	Intermediate	0.0000	0.9785	0.0215	0.2445	0.7555	0.0000
**droperidol**	Intermediate	0.0000	0.8275	0.1725	0.0000	1.0000	0.0000
**pimozide**	Intermediate	0.0130	0.9870	0.0000	0.9350	0.0650	0.0000
**clozapine**	Intermediate	0.0000	1.0000	0.0000	1.0000	0.0000	0.0000
**diltiazem**	Low	1.0000	0.0000	0.0000	0.0000	1.0000	0.0000
**mexiletine**	Low	0.0080	0.9920	0.0000	0.1075	0.8925	0.0000
**ranolazine**	Low	0.0000	0.4775	0.5225	0.0000	0.5865	0.4135
**verapamil**	Low	0.2740	0.7260	0.0000	0.0000	0.7845	0.2155
**metoprolol**	Low	0.0000	0.0570	0.9430	0.0000	1.0000	0.0000
**tamoxifen**	Low	0.0000	1.0000	0.0000	0.9525	0.0475	0.0000
**loratadine**	Low	1.0000	0.0000	0.0000	0.4245	0.5755	0.0000
**nifedipine**	Low	1.0000	0.0000	0.0000	1.0000	0.0000	0.0000
**nitrendipine**	Low	1.0000	0.0000	0.0000	1.0000	0.0000	0.0000

For each of the 28 drugs, the fractions of its 2000 samples classified into the three CiPA TdP risk categories using the MICE model are given by p(Low), p(Inter), and p(High) respectively. Simulation procedure is the same as Table 11.

Table 20. CiPA drugs' probabilities of being classified into the 3 TdP Risk categories based on LOOCV using Bnet.

Drug	True Risk	Manual (Crumb et al.)			HTS (Site 6)		
		p(Low)	p(Inter)	p(High)	p(Low)	p(Inter)	p(High)
**quinidine**	High	0.0000	0.0000	1.0000	0.0035	0.7150	0.2815
**bepridil**	High	0.0045	0.8375	0.1580	0.0220	0.9780	0.0000
**dofetilide**	High	0.0000	0.0000	1.0000	0.0030	0.9970	0.0000
**sotalol**	High	0.0000	0.8555	0.1445	0.0005	0.9995	0.0000
**ibutilide**	High	0.0000	0.0000	1.0000	0.0000	0.0000	1.0000
**azimilide**	High	0.0000	0.9990	0.0010	0.0000	0.0000	1.0000
**disopyramide**	High	0.0045	0.9955	0.0000	0.0000	0.0295	0.9705
**vandetanib**	High	0.0000	0.0000	1.0000	0.0000	0.1800	0.8200
**chlorpromazine**	Intermediate	0.0000	1.0000	0.0000	0.1915	0.8085	0.0000
**cisapride**	Intermediate	0.0000	0.9940	0.0060	0.0120	0.9880	0.0000
**terfenadine**	Intermediate	0.0000	0.0145	0.9855	0.2060	0.7915	0.0025
**ondansetron**	Intermediate	0.0005	0.9905	0.0090	0.1620	0.8380	0.0000
**risperidone**	Intermediate	0.0000	1.0000	0.0000	0.0015	0.9985	0.0000
**domperidone**	Intermediate	0.9975	0.0025	0.0000	0.0000	0.0000	1.0000
**clarithromycin**	Intermediate	0.0075	0.9925	0.0000	0.0000	1.0000	0.0000
**astemizole**	Intermediate	0.0000	0.9415	0.0585	0.0000	1.0000	0.0000
**droperidol**	Intermediate	0.0000	0.9800	0.0200	0.0000	1.0000	0.0000
**pimozide**	Intermediate	0.6760	0.3240	0.0000	0.3100	0.6900	0.0000
**clozapine**	Intermediate	0.0000	1.0000	0.0000	0.1960	0.8040	0.0000
**diltiazem**	Low	1.0000	0.0000	0.0000	0.0010	0.9990	0.0000
**mexiletine**	Low	1.0000	0.0000	0.0000	1.0000	0.0000	0.0000
**ranolazine**	Low	0.0025	0.9940	0.0035	0.0125	0.9700	0.0175
**verapamil**	Low	0.9500	0.0500	0.0000	0.0000	0.4540	0.5460
**metoprolol**	Low	0.5315	0.4685	0.0000	0.1990	0.8010	0.0000
**tamoxifen**	Low	0.0000	1.0000	0.0000	0.0000	1.0000	0.0000
**loratadine**	Low	0.0000	1.0000	0.0000	0.0000	1.0000	0.0000
**nifedipine**	Low	1.0000	0.0000	0.0000	0.9965	0.0035	0.0000
**nitrendipine**	Low	0.9860	0.0140	0.0000	0.1840	0.8160	0.0000

For each of the 28 drugs, the fractions of its 2000 samples classified into the three CiPA TdP risk categories using the metric Bnet are given by p(Low), p(Inter), and p(High) respectively. Simulation procedure is the same as Table 11.

8. Conclusions

Over two pre-specified validation datasets, the candidate CiPA model and metric predefined by training data meet all pre-specified performance measures, and outperform all alternative metrics. This suggests the CiPAORdv1.0 model and qNet/*torsade metric* score proarrhythmia metric are fit for regulatory use under the new CiPA cardiac safety assessment paradigm.

References

(1) Chang, K.C. *et al.* Uncertainty Quantification Reveals the Importance of Data Variability and Experimental Design Considerations for in Silico Proarrhythmia Risk Assessment. *Frontiers in Physiology* **8**, (2017).

(2) Dutta, S. *et al.* Optimization of an In silico Cardiac Cell Model for Proarrhythmia Risk Assessment. *Front Physiol* **8**, 616 (2017).

(3) Li, Z. *et al.* Improving the In Silico Assessment of Proarrhythmia Risk by Combining hERG (Human Ether-a-go-go-Related Gene) Channel-Drug Binding Kinetics and Multichannel Pharmacology. *Circ Arrhythm Electrophysiol* **10**, e004628 (2017).

(4) Zhou, Z. *et al.* Properties of HERG channels stably expressed in HEK 293 cells studied at physiological temperature. *Biophys J* **74**, 230-41 (1998).

(5) Crumb, W.J., Jr., Vicente, J., Johannesen, L. & Strauss, D.G. An evaluation of 30 clinical drugs against the comprehensive in vitro proarrhythmia assay (CiPA) proposed ion channel panel. *J Pharmacol Toxicol Methods*, (2016).

(6) Wible, B.A., Kuryshev, Y.A., Smith, S.S., Liu, Z. & Brown, A.M. An ion channel library for drug discovery and safety screening on automated platforms. *Assay Drug Dev Technol* **6**, 765-80 (2008).

(7) Kuryshev, Y.A., Brown, A.M., Duzic, E. & Kirsch, G.E. Evaluating state dependence and subtype selectivity of calcium channel modulators in automated electrophysiology assays. *Assay Drug Dev Technol* **12**, 110-9 (2014).

(8) Soetaert, K., Petzoldt, T. & Setzer, R.W. Solving Differential Equations in R: Package deSolve. *J Statistical Software* **33**, 1-25 (2010).

(9) Soetaert, K. & Petzoldt, T. Inverse modelling, sensitivity and monte carlo analysis in R using package FME. *J Statistical Software* **33**, 1-28 (2010).

(10) Redfern, W.S. *et al.* Relationships between preclinical cardiac electrophysiology, clinical QT interval prolongation and torsade de pointes for a broad range of drugs: evidence for a provisional safety margin in drug development. *Cardiovas Res* **58**, 32-45 (2003).

(11) Bevilacqua, R., Benassi, C.A., Largajolli, R. & Veronese, F.M. Psychoactive butyrophenones: binding to human and bovine serum albumin. *Pharmacol Res Commun* **11**, 447-54 (1979).

(12) Kramer, J. *et al.* MICE models: superior to the HERG model in predicting Torsade de Pointes. *Sci Rep* **3**, 2100 (2013).

(13) Jankovic, S.M. Pharmacokinetics of selective beta1-adrenergic blocking agents: prescribing implications. *Expert Opin Drug Metab Toxicol* **10**, 1221-9 (2014).

(14) Wieselgren, I., Lundborg, P., Sandberg, A., Olofsson, B. & Bergstrand, R. Pharmacokinetic and pharmacodynamic evaluation of metoprolol controlled release (CR/ZOK) 50 mg in young subjects. *J Clin Pharmacol* **30**, S28-32 (1990).

(15) Sing, T., Sander, O., Beerenwinkel, N. & Lengauer, T. ROCR: visualizing classifier performance in R. *Bioinformatics* **21**, 3940-1 (2005).

(16) Mirams, G.R. *et al.* Simulation of multiple ion channel block provides improved early prediction of compounds' clinical torsadogenic risk. *Cardiovasc Res* **91**, 53-61 (2011).

(17) Lancaster, M.C. & Sobie, E.A. Improved Prediction of Drug-Induced Torsades de Pointes Through Simulations of Dynamics and Machine Learning Algorithms. *Clin Pharmacol Ther* **100**, 371-9 (2016).

(18) Mistry, H.B. Complex versus simple models: ion-channel cardiac toxicity prediction. *PeerJ* **6**, e4352 (2018).
